# Two simple methods to improve the accuracy of the genomic selection methodology

**DOI:** 10.1186/s12864-023-09294-5

**Published:** 2023-04-26

**Authors:** Osval A. Montesinos-López, Kismiantini, Abelardo Montesinos-López

**Affiliations:** 1 Facultad de Telemática, Universidad de Colima, 28040 Colima, México; 2 Statistics Study Program, Universitas Negeri Yogyakarta, 55281 Yogyakarta, Indonesia; 3 Centro Universitario de Ciencias Exactas e Ingenierías (CUCEI), Universidad de Guadalajara, Jalisco 44430 Guadalajara, México

**Keywords:** Genomic prediction, Genomic selection, Reformulation, Binary classification, Postprocessing method

## Abstract

**Background:**

Genomic selection (GS) is revolutionizing plant and animal breeding. However, still its practical implementation is challenging since it is affected by many factors that when they are not under control make this methodology not effective. Also, due to the fact that it is formulated as a regression problem in general has low sensitivity to select the best candidate individuals since a top percentage is selected according to a ranking of predicted breeding values.

**Results:**

For this reason, in this paper we propose two methods to improve the prediction accuracy of this methodology. One of the methods consist in reformulating the GS (nowadays formulated as a regression problem) methodology as a binary classification problem. The other consists only in a postprocessing step that adjust the threshold used for classification of the lines predicted in its original scale (continues scale) to guarantee similar sensitivity and specificity. The postprocessing method is applied for the resulting predictions after obtaining the predictions using the conventional regression model. Both methods assume that we defined with anticipation a threshold, to divide the training data as top lines and not top lines, and this threshold can be decided in terms of a quantile (for example 80%, 90%, etc.) or as the average (or maximum) of the performance of the checks.

In the reformulation method it is required to label as one those lines in the training set that are equal or larger than the specified threshold and as zero otherwise. Then we train a binary classification model with the conventional inputs, but using the binary response variable in place of the continuous response variable. The training of the binary classification should be done to guarantee a more similar sensitivity and specificity, to guarantee a reasonable probability of classification of the top lines.

**Conclusions:**

We evaluated the proposed models in seven data sets and we found that the two proposed methods outperformed by large margin the conventional regression model (by 402.9% in terms of sensitivity, by 110.04% in terms of F1 score and by 70.96% in terms of Kappa coefficient, with the postprocessing methods). However, between the two proposed methods the postprocessing method was better than the reformulation as binary classification model. The simple postprocessing method to improve the accuracy of the conventional genomic regression models avoid the need to reformulate the conventional regression models as binary classification models with similar or better performance, that significantly improve the selection of the top best candidate lines. In general both proposed methods are simple and can easily be adopted for use in practical breeding programs, with the guarantee that will improve significantly the selection of the top best candidates lines.

**Supplementary Information:**

The online version contains supplementary material available at 10.1186/s12864-023-09294-5.

## Introduction

Genomic selection (GS) proposed by [1] around 20 year ago is a predictive methodology. This methodology is very promising since candidates’ individuals can be selected before they are planted in the field, based on predictions resulting of a statistical machine learning model that was trained with a reference population containing phenotypic and genotypic information, but for the target population only is required genotypic information [2].

The name GS is due to the use of high density markers with a good coverage of the whole genome [1]. The GS methodology is attractive since allows: (a) saving time needed for variety development by reducing the cycle length, (b) to substantially increase the selection intensity, which is key for capturing greater gain per unit time, (c) to select traits that are complex to measure, and (d) to improve the accuracy of the selection process, (e) selecting individuals before they are planted in the field, (f) with significant probability selecting superior genotypes with high precision, (g) reducing costs, in part by saving the resources required for extensive phenotyping.

The GS methodology is very promising since can reduce generation intervals for the development of new cultivars and guarantee a reasonable prediction accuracy in the selection process [3]. For these reasons, it had been implemented for the development of many crops like maize, wheat, cassava, chickpea, rice, groundnut, soybean among others [4–7]. Nevertheless, the real, practical application of the GS methodology is complex since many factors affects its performance which significantly increase its uncertainty.

Some factors that affect the accuracy of the GS methodology are: (a) the degree of relatedness between individuals in the training and testing set [8], (b) the heritability of the trait of interest, (c) the size of the testing (target) and training (reference) sets [9], (d) the prediction goal for example untested lines in tested environments, tested lines in tested environments, untested lines in untested environments or tested lines in untested environments [9] ( e) the statistical machine learning algorithm of choice for making the predictions, (f) marker density, (g) relatedness between training and testing individuals [9], (h) population structure, etc. [10].

Some studies that provide specific details for the successful implementation of GS are those in [9, 11]. The first one explain with a lot of details in the context of cereal breeding many factors that affect the accuracy of the GS methodology, as well as many statistical models that are popular in GS, also explain with details in which stages of the breeding process this methodology can be implemented. The second one, made and excellent review about genomic selection and propose how to integrate many tools to improve its efficiency. These authors propose an integrated breeding platform for GS. The platform involves various breeding technologies, including doubled haploid (DH) technology, speed breeding, decision support tools (Genotyping, phenotyping, germplasm and envirotyping), seed DNA-based genotyping, genome editing, and transgenosis. These authors point out that in addition to trying to improve genomic prediction accuracy it is of paramount importance to integrate GS with other breeding technologies to be able to shorten the breeding cycle time and in this way increase significantly the efficiency of the GS methodology. Also, in [11] they propose the need of establishing an open-source breeding network for GS, since there is a correlation between the increase in genetic gain and the increase in the available inputs required to implement GS and for this reason it is important sharing various resources including facilities, platforms, and breeding-related data across GS breeding programs to increase the probability of success even in small breeding programs.

However, many of the factors that affect the GS methodology are not easy to optimize since some requires a significant increase in resources and others even with more resources are not easy to optimize. Nevertheless, one factor that is considerable cheap to optimize is related to the statistical machine learning algorithm to use. The GS methodology traditionally implements a regression model for predicting breeding values or phenotypic values, and some of the most popular regression methods are mixed models and its Bayesian counterpart (BayesA, BayesB, …, Bayesian Lasso, etc.), also had been explored many other methods like random forest, deep neural networks, support vector machine (SVM), kernel methods in conjunction of mixed models, Bayesian methods, SVM, etc. [12].

In general, there are not large differences between the performance of many statistical machine learning methods which is supported by the *non-free lunch theorem* that suggest that “all statistical machine learning algorithms perform equally well when their performance is averaged over all possible objective functions or different data sets”. For this reason, still it is predominant the use of mixed models and Bayesian methods not because they are better in terms of prediction performance but mostly because its implementation is easy, since not a complex tunning process is required.

However, even that many statistical machine learning methods had been implemented for GS, still the predictions of the GS methodology in some cases are quite uncertain and not enough for the practical implementation of this methodology. For this reason, in this paper we propose two methods to improve the accuracy of the GS methodology. One method (B Model) reformulate the conventional genomic regression model (R model) as a binary classification problem, that offer the advantage that will increase significantly the sensitivity to detect the best top lines.

The second method consist in a simple postprocessing method (RO model) to improve the sensitivity to select the top lines. This RO model uses the predictions resulting of the R model which are continues (in the original scale) and with a postprocessing is obtained an optimal threshold to improve the classification of the top lines. For this reason, the RO model only help to optimize the use of conventional genomic prediction models, but can significantly improve the selection of top lines.

Both proposed methods require a threshold as a function of the trait of interest, to divide the training data as top lines and not top lines. This threshold can be defined in terms of a quantile (for example 80%, 90%, etc.) or as the average (or maximum) performance of the checks. Then under the reformulation approach (B model) we define a binary response variable with one for those top lines that are equal or larger than the specified threshold and as zero otherwise. Then under this model B, it is trained a binary classification model, in this case we used a Bayesian threshold genomic best linear unbiased predictor (TGBLUP) model, with the conventional inputs but using the binary response variable in place of the continuous response variable. The training process is implemented in such a way to guarantee at least similar sensitivity and specificity to guarantee a reasonable classification of the top lines.

The context in which the two proposed methods are helpful is when the selection process of the top lines is done regarding the performance of checks (best check, average of checks, certain percentage above the best or average checks, top percent of the training set, etc.). That is, when the selection process is done comparing the continues predicted values of candidate lines regarding a reference check (that is used as threshold) and those lines that have better performance than this reference check (Threshold) are selected for the next generation.

The proposed methods come to fix the problem that conventional genomic prediction models (formulated as a regression models) had, that when selecting the top lines regarding a threshold has low sensitivity to select the top lines. This low sensitivity to select the top lines is due to the fact, that only a small subset of the lines used during the training process are top lines. In this study, we used seven public data sets previously reported in other publications to illustrate the proposed methods. For benchmarking purposes, we compare the results of the two proposed methods with those of the conventional genomic prediction model formulated as a regression model.

## Materials and methods

### Data sets

We used seven data sets for evaluating the proposed methods in this study. A summary of the seven data sets is provided in Table 1.

**Table 1:** Summary of the seven data sets. GBS denotes the genotyping-by-sequencing technology and MAF denotes minor allele frequency

Data	Acronym	Year	No. lines	No. markers	Markers Technology	MAF
*Dataset1*	*EYT_1*	*2013–2014*	*766*	2,038	*GBS*	*0.05*
*Dataset2*	*EYT_2*	*2014–2015*	*775*	2,038	*GBS*	*0.05*
*Dataset3*	*EYT_3*	*2015–2016*	*964*	2,038	*GBS*	*0.05*
*Dataset4*	*Wheat_1*	2013-14/2014-15	*1301*	78,606	*GBS*	*0.05*
*Dataset5*	*Wheat_4*	2016-17/2017-18	*1388*	78,606	*GBS*	*0.05*
*Dataset6*	*Wheat_5*	2017-18/2018-19	*1398*	78,606	*GBS*	*0.05*
*Dataset7*	*Wheat_6*	2018-19/2019-20	*1277*	78,606	*GBS*	*0.05*

Next are described with more details the seven data sets.

#### Datasets 1–3. Elite wheat yield trial (EYT) years 2013–2014 (EYT_1) and 2014–2015 (EYT_2), 2015–2016 (EYT_3)

These are public data sets used by many authors [13–15]. These three datasets were collected by the Global Wheat Program (GWP) of the International Maize and Wheat Improvement Center (CIMMYT) and belong to elite yield trials (EYT) established in four different cropping seasons with 4 or 5 environments in each. The lines involved in this study correspond to years 2013–2014 (Dataset1; EYT_1), 2014–2015 (Dataset2; EYT_2) and 2015–2016 (Dataset3; EYT_3). The EYT datasets 1, 2 and 3 contain 776, 775 and 964 lines, respectively. The experimental design used was an alpha-lattice design and the lines were sown in 39 trials, each covering 28 lines and two checks in six blocks with three replications.

In these datasets, several traits were available for some environments and lines. In this study we included four traits that were measured for each line in each environment: days to heading (DTHD, number of days from germination to 50% spike emergence), days to maturity (DTMT, number of days from germination to 50% physiological maturity or the loss of the green color in 50% of the spikes), plant height and grain yield (GY). For full details of the experimental design and how the BLUEs were computed, see [16].

Data sets 2 and 3 were evaluated in 5 environments, but dataset1 was evaluated in only 4. For EYT dataset1, the environments were bed planting with 5 irrigations (Bed5IR), early heat (EHT), flat planting and 5 irrigations (Flat5IR) and late heat (LHT). For EYT dataset2, the environments were bed planting with 2 irrigations (Bed2IR), Bed5IR, EHT, Flat5IR and LHT, while for dataset3, the environments evaluated were Bed2IR, Bed5IR, Flat5IR, flat planting with drip irrigation (FlatDrip) and LHT. It is important to point out that in this study the data used (BLUEs) was across environments that resulted in the following number of lines used in each data set: 766 in in dataset1, 775 in dataset2 and 964 in dataset3.

Genome-wide markers for the 2515 (776 + 775 + 964) lines in the three datasets were obtained using genotyping-by-sequencing (GBS) [17, 18] at Kansas State University using an Illumina HiSeq2500. After filtering, 2,038 markers were obtained from an initial set of 34,900 markers. The imputation of missing markers data was carried out using LinkImpute [19] and implemented in TASSEL [20], version 5. Lines that had more than 50% missing data were removed, and 2,515 lines were used in this study (776 lines in the first dataset, 775 lines in the second dataset, and 964 lines in the third dataset).

#### Datasets 4–7. Wheat data

Also, these datasets are public and had been used in many publications [13, 14]. Spring wheat lines selected for grain yield analyses from CIMMYT first year yield trials (YT) were used as the training population to predict the quality of lines selected from elite yield trials (EYT) for grain yield analyses in a second year. Details of these four data sets are given next: - Wheat_1 (2013-14/2014-15; denoted as dataset4), 1,301 lines from the 2013-14 EYT and 472 lines from the 2014-2015 EYT trial. In this dataset, only the grain yield trait was used. The lines across environments that were used in this study were 1301. - Wheat_4 (2016-17/2017-18; denoted as dataset5), 1,372 lines from the 2016-17 EYT and 567 lines from the 2017-2018 EYT trial. The lines across environments that were used in this study were 1388. - Wheat_5 (2017-18/2018-19; denoted as dataset6), 1,386 lines from the 2017-18 EYT and 509 lines from the 2018-2019 EYT trial. In this dataset, only the grain yield trait was used. The lines across environments that were used in this study were 1398. - Wheat_6 (2018-19/2019-20; denoted as dataset7), 1,276 lines from the 2018-19 EYT and 124 lines from the 2019-2020 EYT trial. More details of these datasets can be found in [21]. The lines across environments that were used in this study were 1277. All the lines were genotyped using genotyping-by-sequencing (GBS; [18]). The TASSEL v.5 (Trait Analysis by Association Evolution and Linkage) GBS pipeline was used to call marker polymorphisms [22], and a minor allele frequency of 0.01 was used for single nucleotide polymorphism (SNP) discovery. The resulting 6,075,743 unique tags were aligned to the wheat genome reference sequence (RefSeq v.1.0) [23] with an alignment rate of 63.98%. After filtering for SNPs with homozygosity >80%, p-value for Fisher’s exact test <0.001 and Chi-square value lower than the critical value of 9.2, we obtained 78,606 GBS markers that passed at least one of those filters. These markers were further filtered for less than 50% missing data, greater than a 0.05 minor allele frequency and less than 5% heterozygosity in all the datasets. Markers with missing data were imputed using the ‘expectation-maximization’ algorithm in the ‘R’ package rrBLUP [24].

### Statistical methods

#### Model R

Model R, is the Bayesian best linear unbiased predictor (GBLUP) model, that is formulated as a regression problem. This model is given next:

1 $${Y}_{i}=\mu +{g}_{i}+{\epsilon}_{i}$$

Where ${Y}_{i}$ denotes the continues response variable measured in the ith line, $\mu$ is a general mean or intercept. ${g}_{j},$ $i=1,\dots ,J$, denotes the random effect of ith genotype, and ${\epsilon}_{i}$ is the random error component of ith genotype distributed as an independent normal random variable with mean 0 and variance ${\sigma }^{2}$. It is assumed that $\varvec{g}={\left({g}_{1},\dots ,{g}_{J}\right)}^{T}\sim {N}_{J}\left(0,{\sigma }_{g}^{2}\varvec{G}\right)$, where $\varvec{G}$ is a linear kernel known as genomic relationship matrix computed according with the method of [25]. This model was implemented in the R statistical software [26] with the BGLR library of [27].

Since we are interested in selecting the top lines for each trait, the threshold, ${Y}_{\tau }$, (where ${Y}_{\tau }$is the empirical quantile $\tau$ of training response values (${Y}_{1}$,…,${Y}_{{n}_{tr}}$), we used $\tau =0.8$, but any other value between 0 and 1 can be used) is used for the classification of the lines as top lines (denotes as 1; if $\widehat{{Y}_{i}}>{Y}_{\tau }$, for $i=1,\dots ,{n}_{tst}$) and not top lines (denotes as 0; if $\widehat{{Y}_{i}}<{Y}_{\tau }, \text{f}\text{o}\text{r} i=1,\dots ,{n}_{tst}$). For this reason, after we obtain the continuous predictions with this model those lines with predicted values larger than this threshold, ${Y}_{\tau },$were classified as top lines and those with predicted values less than the threshold were classified as not top lines.

#### Model B

Model B is the threshold Bayesian probit binary model (TGBLUP) that assumes that conditioned to ${\varvec{g}}_{i}$ (covariates of dimension $J$), ${Y}_{bi}$ is a random variable that takes values ​​0 and 1, with the following probabilities:

2 $$P\left({Y}_{bi}=1|{g}_{i}\right)={\Phi }\left( {{\upbeta }}_{0}+{g}_{i}\right)=P\left({l}_{i}>0\right)$$

where ${\beta }_{0}$is an intercept parameter, ${g}_{i}$ denotes the random effect of the ith genotype distributed exactly as was defined in model (1), and ${l}_{i}={{\upbeta }}_{0}+{g}_{i}{+\epsilon}_{i}$ is the underlying or latent continuous normal process that gives rise to the observed categories (top lines and not top lines), where ${\epsilon}_{i}$ is a normal random variable for errors with mean 0 and variance 1. The values of ${l}_{i}$ are called “liabilities” [28, 29]. The binary categorical phenotypes in model (2) are generated from the underlying phenotypic values, ${l}_{i}$, as follows: ${y}_{bi}=0$if $-{\infty }{<l}_{i}<0, \text{o}\text{t}\text{h}\text{e}\text{r}\text{w}\text{i}\text{s}\text{e}$ ${y}_{bi}=1.$ Given that model (2) is formulated under a Bayesian framework for this reason this model assumes a flat prior distribution for ${\beta }_{0}$ ($f\left({\beta }_{0} \right)\propto 1$). The TGBLUP model was implemented in the BGLR package of [27] in the R statistical software [26].

**Fig. 1:**
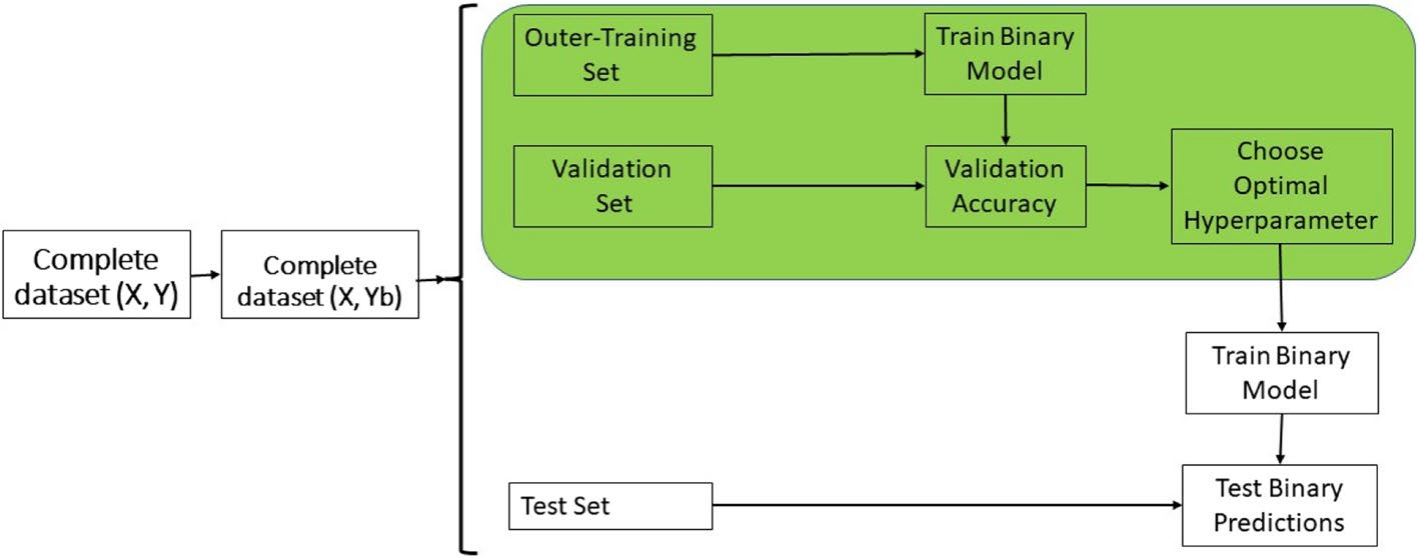
Schematic representation of the training process under model B. **X** denotes the input (markers and other covariates) data, **Y** the continuous response variable and **Y**b the binary response variable resulting of transforming **Y** to a binary response variable. The final predictions are done as 1 for top lines and 0 as not top lines

Figure 1 provides a representation of the steps required for the training process under model B. These steps are described next:

- Step 1: First transform the continuous response variable to a binary response variable using the same threshold (for example average performance of checks) mentioned before in the R model, but now using the observed original response variable. That is, when the values of the continues traits are larger than the specific threshold, then is assigned a value of one (1 = top line) otherwise a zero (0 = not top lines).
- Step 2: First split the data in inner-training, validation and test set.
- Step 3. Train model B (Classification model) with the inner-training set,
- Step 4. Using the trained model B (in step 3) with the validation set compute the predicted probabilities, ${\widehat{P}}_{Val,l}$ for $i=1,\dots ,{n}_{val},$for the validation set and after use these predicted probability values to estimate the metrics of classification accuracy to select the optimal probability threshold ${(\tau }_{0})$.
- Step 5. Then select the optimal hyperparameter, that is, the probability threshold (${\tau }_{0})$ that minimize the average of the squared difference between the sensibility and specificity.
- Step 6. Then with the whole training set (inner-training + validation) refit model B and produce probability predictions for the testing set, that is,${\widehat{P}}_{Test,l}$ for $i=1,\dots ,{n}_{Test},$for the testing set.
- Step 6. Then with the optimal probability threshold (${\tau }_{0})$ of Step 5 and the predicted probabilities of the testing set of Step 6, we classified the lines as.

If ${\widehat{P}}_{Test,l}>{\tau }_{0}$ the line is classified at top line (1), otherwise as not top line (0).

Details of the steps are given in Fig. 1.

#### Model RO

**Fig. 2:**
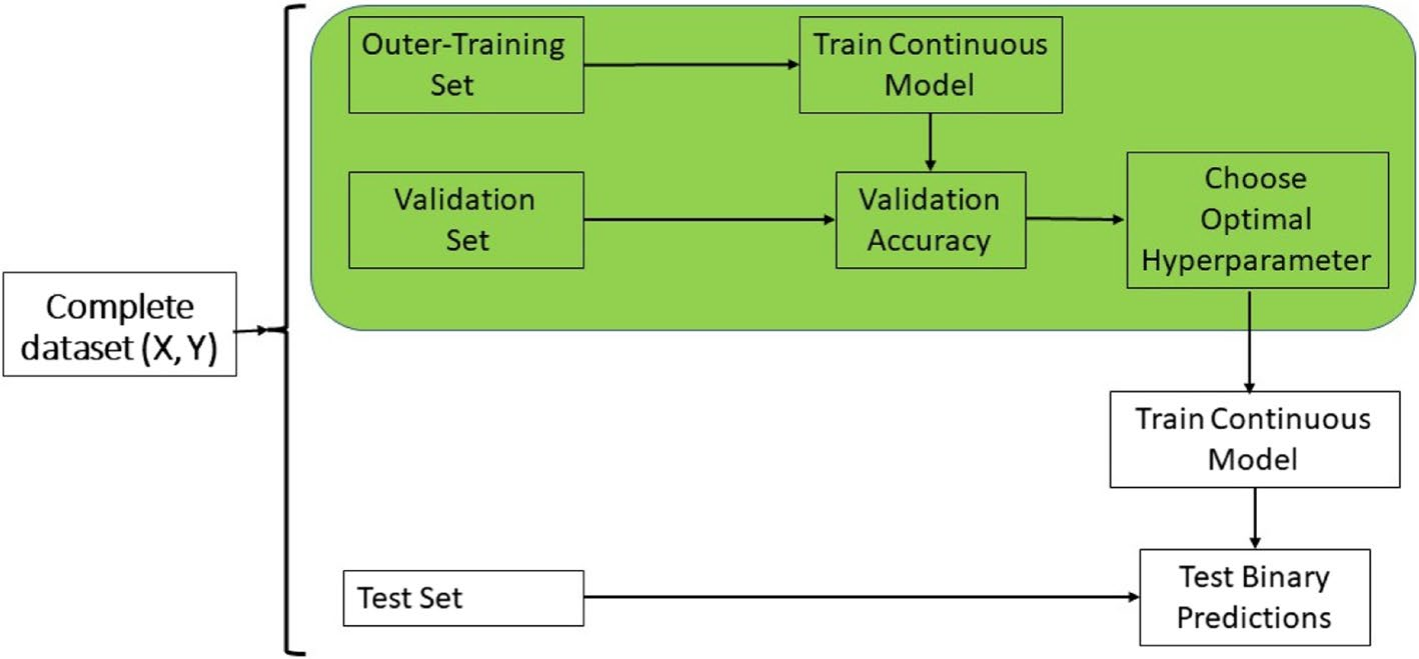
Schematic representation of the training process under model RO. **X** denotes the input (markers and other covariates) data and **Y** the continuous response variable. The final predictions are done as 1 for top lines and 0 as not top lines

The RO acronym denotes regression optimum, this model use model R for the training process to adjust the threshold (computed with the 80% quantile of the response variable of the training set or with the average performance of checks) to be able to guarantee a similar sensitivity and specificity. An schematic representation of the steps involved in the training process of model RO are given in Fig. 2. These steps are described next:

- Step 1: First split the data in inner-training, validation and test set.
- Step 2. Train model R with the inner-training set using the original response variable.
- Step 3. Using the trained model R (in step 2) with the validation set compute the predicted continues values, ${\widehat{Y}}_{Val,l}$ for $i=1,\dots ,{n}_{val},$for the validation set, and after use this predicted values to estimate the metrics of classification accuracy to select the optimal probability threshold ${(\tau }_{0})$.
- Step 4. Then select the optimal hyperparameter, that is, the probability threshold (${\tau }_{0})$, with which is computed optimal threshold (${Y}_{\tau 0})$ that minimize the average of the squared difference between the sensibility and specificity.
- Step 5. Then with the whole training set (inner-training + validation) refit model R, and with this refitted model compute the predicted values of the testing set, that is, ${\widehat{Y}}_{Test,l}$ .
- Step 6. Then with the optimal threshold (${Y}_{\tau 0})$ computed in Step 4 and the predicted values of the test set in Step 5, we classified the lines as

If ${\widehat{Y}}_{Test,l}>{Y}_{\tau 0}$ the line is classified at top line (1), otherwise as not top line (0).

In Fig. 2 can be appreciated the use of model R during the training process of model RO.

Note that this optimal modified rule can be expressed in terms of the traditional threshold values (${Y}_{\tau }$). This is because classifying a line as top $\left(\widehat{{Y}_{i}}>{Y}_{\tau 0}\right)$ is equivalent to classifying a line if ${\widehat{Y}}_{i}^{*}>{Y}_{\tau }$ where ${\widehat{Y}}_{i}^{*}=\widehat{{Y}_{i}}($ ${Y}_{\tau }/{Y}_{\tau 0})$ is the modified predicted values or adjusted predicted value that guarantee similar sensitivity and specificity.

Finally, since with the three models evaluated (R, RO and B) the predictions are in terms of zeros (not top lines) and ones (top lines) were computed classification metrics for evaluating the prediction accuracy for the testing sets.

### Metrics for evaluation of prediction performance

In each of the seven data sets, one outer-fold cross validation and one inner-fold cross validation was implemented [12]. The outer-fold cross-validation was implemented for evaluating the prediction accuracy in out-of-sample data using 5 fold cross-validation, while the inner-cross-validation to tune the probability threshold hyperparameter under models B and RO and this was implemented with 10 fold cross-validation (See Figs. 1 and 2). Under the outer-5-fold cross validation the model was trained with four folds and the remaining was used as testing set until each of the 5 folds were used as testing set once. For sure the testing set was not used for training only for evaluating the prediction accuracy. The average of the 5 testing sets was reported as prediction accuracy with 4 metrics that are explained in the following paragraphs.

Next, the average of the 5-folds was reported as prediction performance using the Kappa coefficient, the Sensitivity, the Specificity and the F1 score. For the implementation of the R model (GBLUP) no tuning was required, but for models B and RO was tuned the probability threshold to guarantee similar sensitivity and specificity. For this reason, under models B and RO where tuning was required the inner-cross validation was implemented using ten folds to optimize the probability thresholds that were selected as the average of the ten folds of the inner cross validation. Then these optimal thresholds were used for the classification of the lines at top lines and not top lines in each testing set (See Figs. 1 and 2).

Then with the outputs (predictions) of three models (R, RO and B) for each testing set were computed the metrics that are described next. The Kappa coefficient (κ) statistic is a chance-corrected method for assessing agreement (rather than association) among raters. Kappa is defined as follows:

$$\kappa =\frac{{P}_{0}-{P}_{e}}{1-{P}_{e}}$$

where ${P}_{0}$ is the agreement between predicted and observed values and it is computed by the $\frac{TP+TN}{N}$, where $TN$ is the number of true negatives, $TP$ is the number of true positives, $FN$ is the number of false negatives, $FP$ is the number of false positives, and $N=TP+TN+FP+FN$; ${P}_{e}$ is the probability of agreement calculated as ${P}_{e}=\frac{TP+FN}{N}\times \frac{TP+FP}{N}+\frac{FP+TN}{N}\times \frac{FN+TN}{N}$. **Sensitivity** is defined as the probability of a positive test, conditioned on truly being positive: TP/ (TP+ FN), whereas **specificity** is defined as the probability of a negative test, conditioned on truly being negative: TN/ (TN+ FP) [10]. **Precision** is the ratio of correctly predicted positive observations to the total predicted positive observations (Precision = TP/(TP+FP)). The higher the precision the lower the false positive rate and for sure the higher the precision the better the prediction accuracy.

The **F1 Score** is the weighted average of Sensitivity and Precision. Therefore, this score takes both false negatives and false positives into account. The F1 score is normally more useful than accuracy, especially in unbalanced data sets class distribution. Accuracy works best if false positives and false negatives have similar cost. If the cost of false negatives and false positives are very different, it’s better to look at both Precision and Sensitivity [30]. To compare two methods (R and B or R and RO or B and RO) the relative efficiencies in terms of F1 score were computed as,

$$R{E}_{Kappa}=\frac{{Kappa}_{y}}{{Kappa}_{z}}$$

where ${Kappa}_{y}$ and ${Kappa}_{z}$ denotes the $Kappa$ coefficient of models y and z, respectively. With y = R, B and z = B, RO. While in terms of sensitivity the RE was computed as:

$$R{E}_{Sensitivity}=\frac{{Sensitivity}_{y}}{{Sensitivity}_{z}}$$

In a similar fashion was computed the RE for the F1 score and specificity. Under the four metrics, if $R{E}_{x}>1,$ with $x=Kappa, Sensitivity, Specificity, F1,$the best prediction performance was obtained using method y, but when $R{E}_{x}<1,$ the best method was z. When $R{E}_{x}=1,$ both methods were equally efficient.

## Results

The results are given in 5 sections one for data set EYT_1, EYT_2, Wheat_5, Wheat_6 and the last one for the results across data sets. In each section we compare the prediction performance between the conventional regression model, R, the reformulation as a binary classification problem, model B, and the adjusted conventional regression model (RO). The comparison is done in terms of four metrics F1 score, Kappa coefficient, sensitivity and specificity. For the remaining data sets the results are given in supplemental material.

### EYT_1

**Fig. 3:**
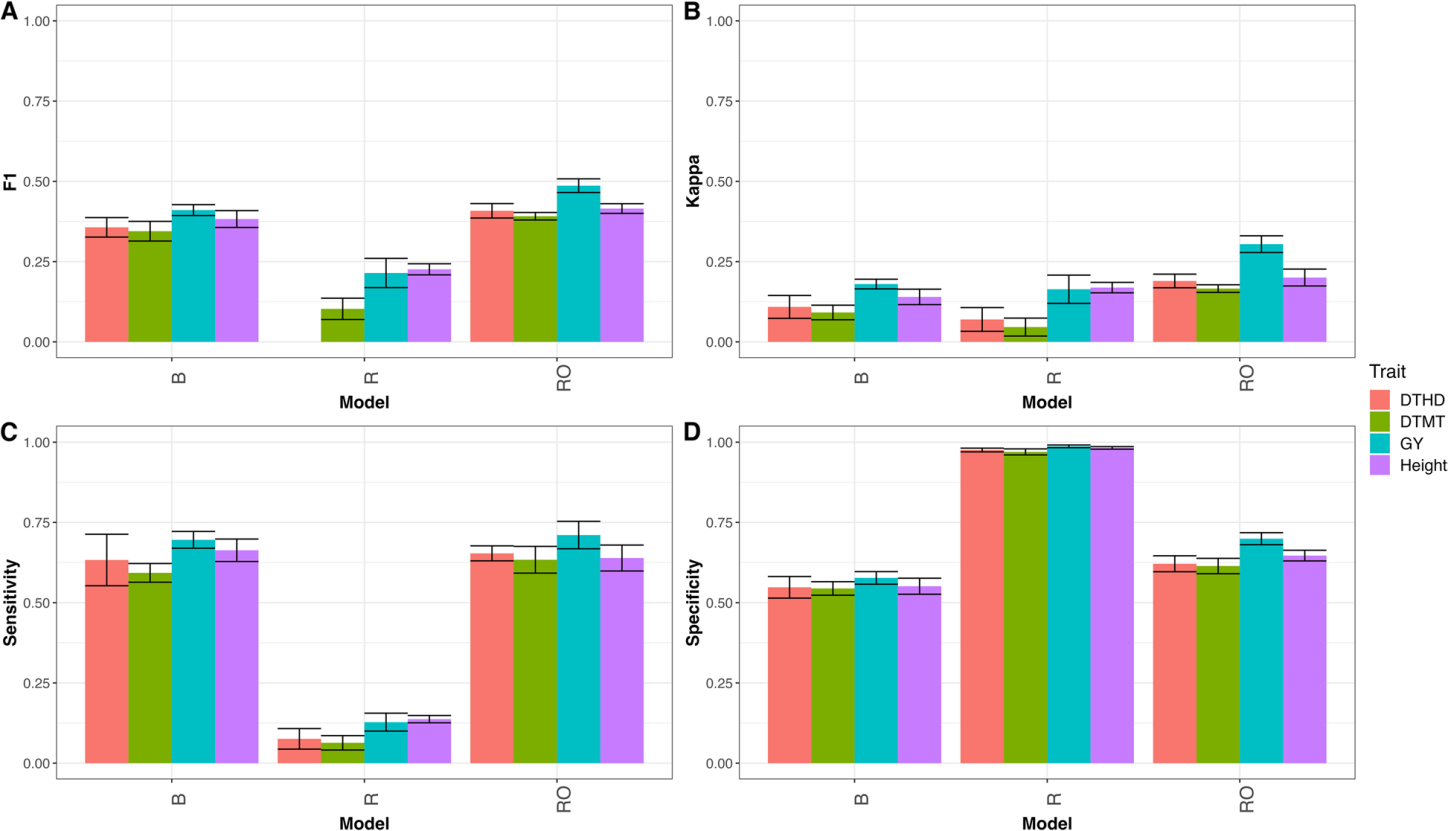
** A** Mean accuracy in terms of F1 score for classification model (B), regression model (R) and regression optimum model (RO) for traits DTHD, DTMT, GY and Height for dataset EYT_1. **B** Mean accuracy in terms of Kappa coefficient for classification model (B), regression model (R) and regression optimum model (RO) for traits DTHD, DTMT, GY and Height for dataset EYT_1. **C** Mean accuracy in terms of sensibility for classification model (B), regression model (R) and regression optimum model (RO) for traits DTHD, DTMT, GY and Height for dataset EYT_1. **D** Mean accuracy in terms of specificity for classification model (B), regression model (R) and regression optimum model (RO) for traits DTHD, DTMT, GY and Height for dataset EYT_1

As can be seen in Fig. 3 the proposed reformulation model, B, has better prediction performance than the conventional regression model, R, but not has better prediction performance than the regression optimum model, RO. In terms of F1 score the accuracy in the four trait was: DTHD (B = 0.357, R = NA, RO = 0.408), DTMT (B = 0.345, R = 0.103, RO = 0.391), GY (B = 0.411, R = 0.215, RO = 0.487) and Height (B = 0.383, R = 0.226, RO = 0.415), that is, in terms of F1 score model B outperformed model R in trait DTMT by 235.0% (RE = 3.350), in trait GY by 91.3% (RE = 1.913) and in trait Height by 69.3% (RE = 1.693). Also, in terms of F1 score model RO outperformed model R in trait DTHD by 12.6%, in trait DTMT by 11.9%, in trait GY by 15.6% and in trait Height by 7.9% for details see appendix Table A1 and Fig. 3A. Also, in terms of Kappa coefficient, model B outperformed model R, but not the RO model, in traits DTHD (B = 0.109, R = 0.070, RO = 0.190), DTMT (B = 0.092, R = 0.046, RO = 0.166) and GY (B = 0.180, R = 0.164, RO = 0.304), only for Height trait model R showed better prediction performance than model B (B = 0.140, R = 0.169, RO = 0.200), in other words in terms of Kappa coefficient model B outperformed model R by 55.9% (RE = 1.559 for trait DTHD), 98.6% (RE = 1.986 for trait DTMT), 9.9% (RE = 1.099 for trait GY). Also, in terms of Kappa coefficient model R outperformed model B in trait Height by 17.2%, while model RO outperformed model B by 42.6% in trait DTHD, by 44.8% in trait DTMT, by 40.8% in trait GY and by 30.1% in trait Height, for details see in appendix Table A1 and Fig. 3B. In terms of sensitivity model B and RO were better than model R in the four traits, but model RO showed better performance than model B in traits DTHD (B = 0.633, R = 0.076, RO = 0.654), DTMT (B = 0.593, R = 0.063, RO = 0.634), GY (B = 0.696, R = 0.128, RO = 0.711) and Height (B = 0.663, R = 0.137, RO = 0.639), that is, in terms of Sensitivity model B outperformed model R by 734.8% (RE = 8.348), 833.6% (RE = 9.336), 4.442% (RE = 5.442) and 383.2% (RE = 4.832%) for traits DTHD, DTMT, GY and Height respectively, on the other hand model RO outperformed model B by 3.2% in trait DTHD, by 6.4% in trait DTMT and by 2.1% in trait GY, but model B outperformed model RO by 3.8% in trait Height. For details see in appendix Table A1 and Fig. 3C. Finally, in terms of specificity model R and RO showed better performance in the four traits DTHD (B = 0.548, R = 0.976, RO = 0.621), DTMT (B = 0.544, R = 0.970, RO = 0.614), GY (B = 0.577, R = 0.987, RO = 0.699) and Height (B = 0.551, R = 0.982, RO = 0.647), that is, model R outperformed model B by 43.8% in trait DTHD, by 43.9% in trait DTMT, by 41.5% in trait GY and by 43.9% in trait Height, while model RO outperformed model B by 11.8% in trait DTHD, by 11.4% in trait DTMT, by 17.5% in trait GY and by 14.8% in trait Height. For details see in appendix Table A1 and Fig. 3D.

### EYT_2

**Fig. 4:**
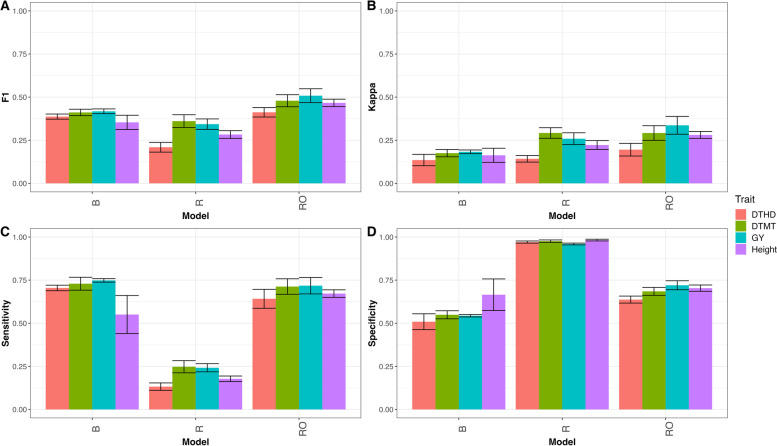
** A** Mean accuracy in terms of F1 score for classification model (B), regression model (R) and regression optimum model (RO) for traits DTHD, DTMT, GY and Height for dataset EYT_2. **B** Mean accuracy in terms of Kappa coefficient for classification model (B), regression model (R) and regression optimum model (RO) for traits DTHD, DTMT, GY and Height for dataset EYT_2. **C** Mean accuracy in terms of sensibility for classification model (B), regression model (R) and regression optimum model (RO) for traits DTHD, DTMT, GY and Height for dataset EYT_2. **D** Mean accuracy in terms of specificity for classification model (B), regression model (R) and regression optimum model (RO) for traits DTHD, DTMT, GY and Height for dataset EYT_2

As can be seen in Fig. 4 the proposed model, RO, outperformed models R and B. In terms of F1 score this was the performance in traits DTHD (B = 0.387, R = 0.209, RO = 0.412), DTMT (B = 0.411, R = 0.361, RO = 0.479), GY (B = 0.418, R = 0.343, RO = 0.508) and Height (B = 0.354, R = 0.283, RO = 0.467), that is, in terms of F1 score model B outperformed model R in trait DTHD by 85.0% (RE = 1.850), in trait DTMT by 14.0% (RE = 1.140), in trait GY by 21.7% (RE = 1.217) and in trait Height by 24.9% (RE = 1.249). While model RO outperformed model R by 6.1% in trait DTHD, by 14.2% in trait DTMT, by 17.8% in trait GY, by 24.2% in trait Height. For details see appendix Table A2 and Fig. 4A. Also, in terms of Kappa coefficient, model R and RO outperformed model B in traits DTHD (B = 0.135, R = 0.142, RO = 0.195), DTMT (B = 0.175, R = 0.292, RO = 0.292), GY (B = 0.183, R = 0.259, RO = 0.337) and Height (B = 0.163, R = 0.222, RO = 0.281), in other words, in terms of Kappa coefficient model R outperformed model B by 5.1% for trait DTHD, 40.1% for trait DTMT, 29.3% for trait GY and 26.8% for trait Height. While model RO outperformed model B by 30.8% in trait DTHD, by 40.0% in trait DTMT, by 45.6% in trait GY and by 42.0% in trait Height. For details see in appendix Table A2 and Fig. 4B. In terms of sensitivity model B was better than models R and RO in traits DTHD (B = 0.705, R = 0.133, RO = 0.642), DTMT (B = 0.729, R = 0.248, RO = 0.713), GY (B = 0.748, R = 0.242, RO = 0.718) and Height (B = 0.551, R = 0.178, RO = 0.672), that is, in terms of Sensitivity model B outperformed model R by 431.4% (RE = 5.314), 194.2% (RE = 2.942), 209.4% (RE = 3.094) and 209.5% (RE = 3.095%) for traits DTHD, DTMT, GY and Height respectively. Also, model B outperformed model RO by 9.8% (RE = 1.098), 2.3% (RE = 1.023), 4.3% (RE = 1.043) for traits DTHD, DTMT and GY respectively, only in trait Height the model RO outperformed B model by 18.1%, see details in appendix Table A2 and Fig. 4C. Finally, in terms of specificity models R and RO showed better performance in the four traits DTHD (B = 0.509, R = 0.971, RO = 0.637), DTMT (B = 0.549, R = 0.976, RO = 0.685), GY (B = 0.543, R = 0.960, RO = 0.720) and Height (B = 0.665, R = 0.982, RO = 0.704), that is, model R outperformed model B by 47.6% in trait DTHD, by 43.7% in trait DTMT, by 43.4% in trait GY and by 32.3% in trait Height. While model RO outperformed model B by 20.1% in trait DTHD, by 19.8% in trait DTMT, by 24.6% in trait GY and by 5.4% in trait Height for details see in appendix Table A2 and Fig. 4D.

#### Wheat_5

**Fig. 5:**
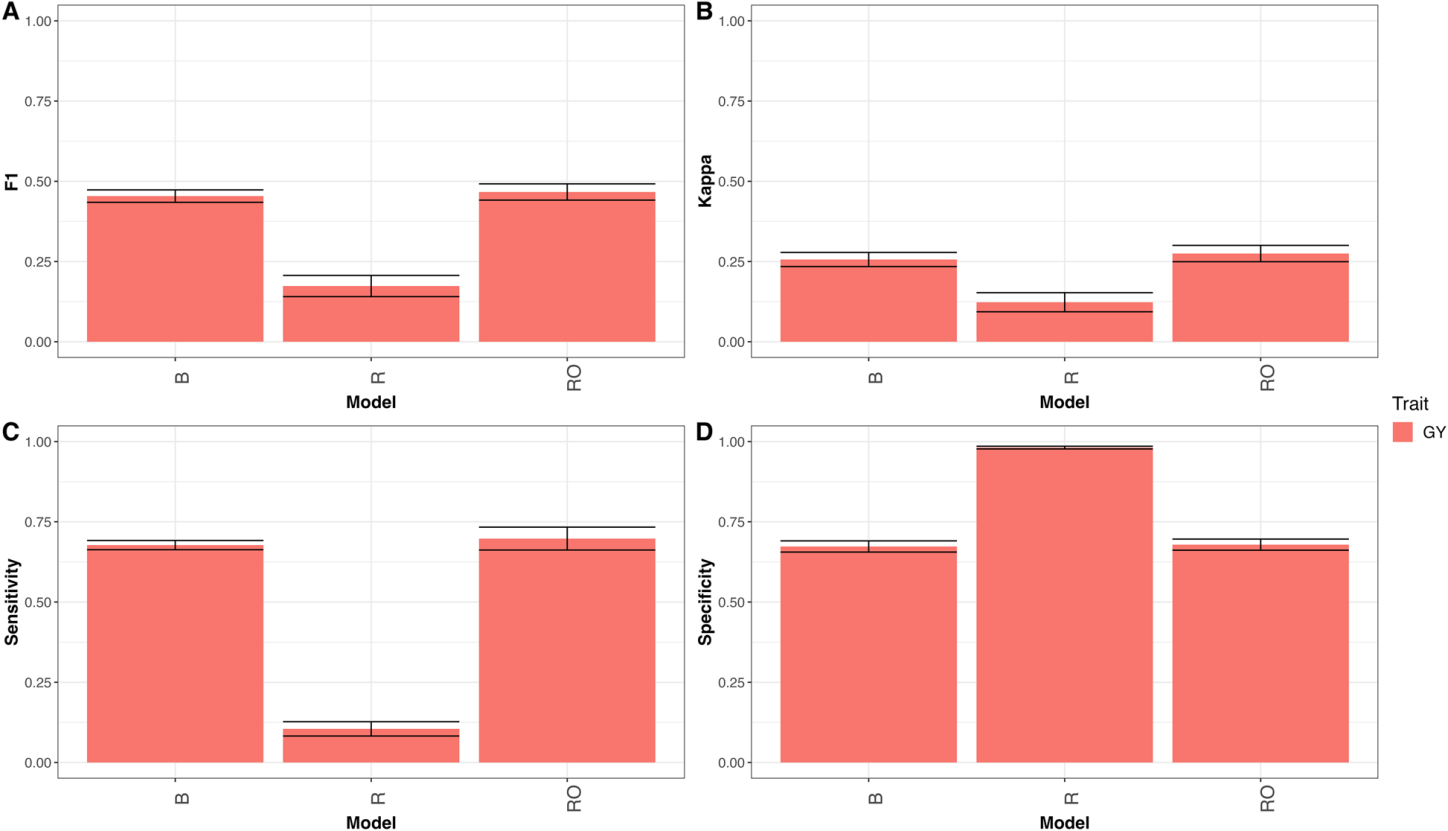
** A** Mean accuracy in terms of F1 score for classification model (B), regression model (R) and regression optimum model (RO) for traits GY for dataset Wheat_5. **B** Mean accuracy in terms of Kappa coefficient for classification model (B), regression model (R) and regression optimum model (RO) for trait GY for dataset Wheat_5. **C** Mean accuracy in terms of sensibility for classification model (B), regression model (R) and regression optimum model (RO) for trait GY for dataset Wheat_5. **D** Mean accuracy in terms of specificity for classification model (B), regression model (R) and regression optimum model (RO) for trait GY for dataset Wheat_5

As can be seen in Fig. 5 model B, has better prediction performance than model R, but not has better prediction performance than model RO. In terms of F1 score in trait GY (B = 0.454, R = 0.174, RO = 0.467) model B outperformed model R by 161.4% (RE = 2.612) and model RO outperformed model B by 2.7%, for details see appendix Table A3 and Fig. 5A. Also, in terms of Kappa coefficient, model B outperformed model R, but not RO model in trait GY (B = 0.256, R = 0.123, RO = 0.275), in other words, model B outperformed model R by 108.2% (RE = 2.082 for trait GY) and model RO outperformed model B by 6.8% for trait GY, for details see in appendix Table A3 and Fig. 5B. Also for GY trait in terms of sensitivity model B was better than model R, but not of model RO (B = 0.677, R = 0.104, RO = 0.698), that is, in terms of sensitivity model B outperformed model R by 546.4% (RE = 6.464) and model RO outperformed model B by 2.9%, for details see in appendix Table A3 and Fig. 5C. Finally, in terms of Specificity models R and RO showed better performance than B model in GY trait (B = 0.673, R = 0.981, RO = 0.679), that is, model R outperformed model B by 31.4% and model RO outperformed model B by 0.8% in trait GY, for details see in appendix Table A3 and Fig. 5D.

#### Wheat_6

**Fig. 6:**
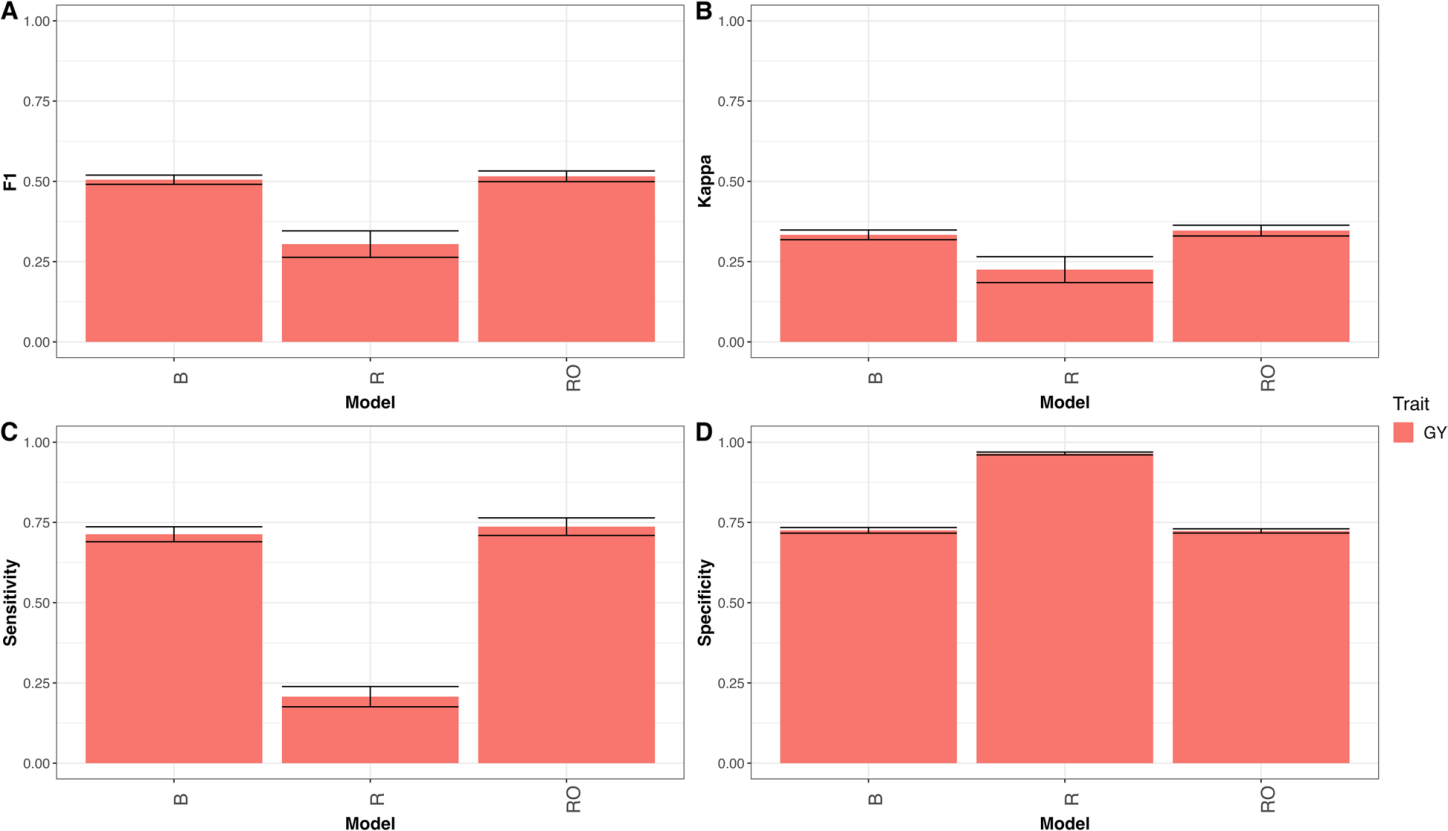
** A** Mean accuracy in terms of F1 score for classification model (B), regression model (R) and regression optimum model (RO) for trait GY for dataset Wheat_6. **B** Mean accuracy in terms of Kappa coefficient for classification model (B), regression model (R) and regression optimum model (RO) for trait GY for dataset Wheat_6. **C** Mean accuracy in terms of sensibility for classification model (B), regression model (R) and regression optimum model (RO) for trait GY for dataset Wheat_6. **D** Mean accuracy in terms of specificity for classification model (B), regression model (R) and regression optimum model (RO) for trait GY for dataset Wheat_6

As can be seen in Fig. 6 model RO has better prediction performance than model R and B. In terms of F1 score this was the prediction performance in trait GY (B = 0.505, R = 0.305, RO = 0.516), that is, in terms of F1 score model B outperformed model R by 65.8% (RE = 1.658), and model RO outperformed model B by 2.1%, for details see appendix Table A4 and Fig. 6A. Also, in terms of Kappa coefficient, model B outperformed model R, but not RO model in trait GY (B = 0.333, R = 0.225, RO = 0.347), in other words model B outperformed model R by 48.1% (RE = 1.481 for trait GY), and model RO outperformed model B by 3.8%, for details see in appendix Table A4 and Fig. 6B. Also, in terms of sensitivity model RO was better than models B and R in GY trait (B = 0.713, R = 0.207, RO = 0.737), that is, in terms of sensitivity model B outperformed model R by 244.1% (RE = 3.441), but model RO outperformed model B by 3.2%, for details see in appendix Table A4 and Fig. 6C. Finally, in terms of specificity the regression model showed better performance that models RO and B in trait GY (B = 0.725, R = 0.965, RO = 0.723), that is, model R outperformed model B by 24.8% and model B outperformed model RO by 0.3% for details see in appendix Table A4 and Fig. 6D.

#### Across datasets

**Fig. 7:**
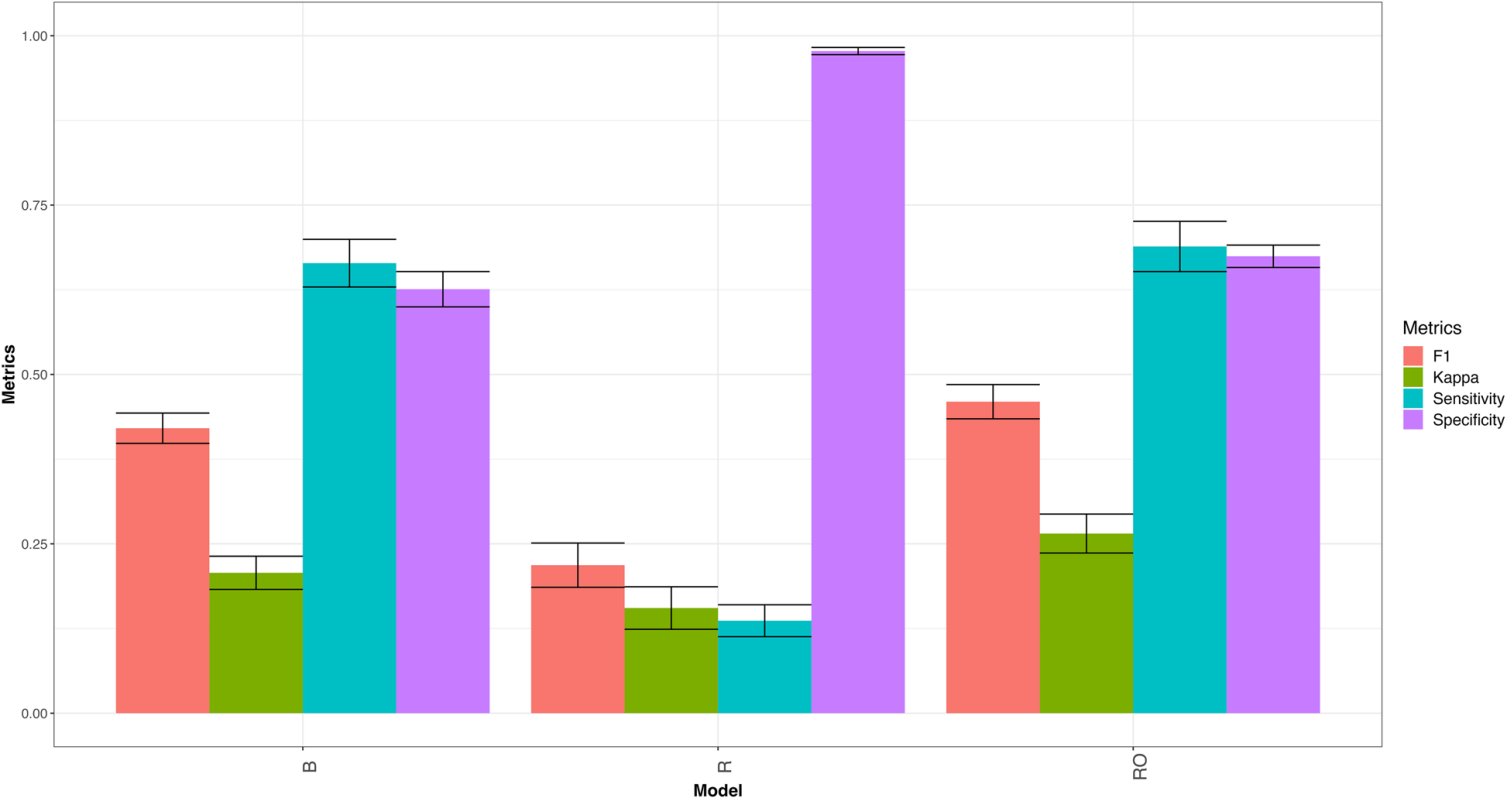
Mean accuracy in terms of F1 score for classification model (B), regression model (R) and regression optimum model (RO) across traits and across datasets

As can be seen in Fig. 7 in general model, RO, has better prediction performance than models R and B. In terms of F1 score across traits and across datasets (B = 0.421, R = 0.219, RO = 0.460), model B outperformed model R by 92.5% (RE = 1.925) and model RO outperformed model B by 8.5%, for details see appendix Table A5 and Fig. 7. Also, in terms of Kappa coefficient, model RO outperformed models R and B across traits and across datasets (B = 0.207, R = 0.155, RO = 0.265), that is, model B outperformed model R by 33.5% (RE = 1.335 for across traits and across datasets), and model RO outperformed model B by 21.9% for details see in appendix Table A5 and Fig. 7. Also, in terms of sensitivity model RO was better than models R and B across traits and across datasets (B = 0.664, R = 0.137, RO = 0.689), that is, model B outperformed model R by 386.6% (RE = 4.866) and model RO was slightly better than model B, for details see in appendix Table A5 and Fig. 7. Finally, in terms of specificity model R showed better performance than models B and RO across traits and across datasets (B = 0.626, R = 0.977, RO = 0.674), that is, model R outperformed model B by 36.0% and model RO outperformed model B by 7.2% for details see in appendix Table A5 and Fig. 7.

## Discussions

The genomic selection methodology that is revolutionizing plant breeding was proposed more than 20 years ago by [1]. It is atractive and efficient since it is a predictive methodology that is able to select candidate individuals before they are planted in the field, thanks to the use of statistical machine learning methods trained with phenotypic and markers data of a reference population and markers of candidate individuals. However, since many factor affects its efficiency and precision (see more details in [10, 11]) for this reason continues the research with the goal for achieving and obtaining greater genetic gain and for improving the production of staple crops to meet the human demand from an increasing global population [11]. For this reason, a successful implementation of the GS needs to guarantee high sensitivity and reasonable specificity in the selection of the top lines. High sensitivity is required to guarantee that the lines selected are the best (those top lines) and reasonable specificity to guarantee that those not top lines are not selected. However, the conventional formulation of the genomic selection methodology as a regression problem, model R, where the model is trained to predict a continues response variable not always guarantee a reasonable sensitivity and specificity since the top lines in the training set are minority. For this reason, the model R most of the time only guarantee high specificity and low sensitivity.

For this reason, in this paper we propose two methods to improve the accuracy of the GS methodology. The first one, reformulate the genomic selection methodology as a classification problem, where those lines in the training set with top performance are allocated to the top class labeled as one and the others are allocated to the zero class. Then in place of training a regression model it is trained a classification model with the binary response variable, just created, with the goal to guarantee at least a similar sensitivity and specificity. While the second one, modified the threshold for classification the continues predictions resulting of the conventional genomic prediction models. The threshold, under this method is optimized to guarantee similar sensitivity and specificity because the conventional prediction model has very low sensitivity and very high specificity. This second method consists on a postprocessing step since we use the continuous predictions produced by the conventional genomic prediction model that is formulated as a regression problem. However, to be able to estimate the optimal threshold each training set was divided in inner-training and validation, and with the inner-training were trained the models and with the validation evaluated the hyperparameter (threshold) from which the optimal threshold was estimated that was used for improving the sensitivity of the classification of the predicted lines. For this reason, the optimal threshold is data dependent. This threshold guarantee similar sensitivity and specificity since was selected in such a way that minimize the average of the squared difference between the sensibility and specificity.

Our results found that the proposed reformulation of the GS methodology, model B, has more power to select the best top lines since outperformed the conventional approach, model R by 92.5, 33.5, 386.6% in terms of F1 score, Kappa and Sensitivity. Also, the proposed reformulation method (model B) decrease significantly the specificity from 97.7% across traits and data sets under the conventional regression formulation to 60% under the proposed reformulation, that is, a reduction in 36% in the specificity. It is important to point out that the conventional regression model has high specificity (97.7% across traits and data set) and for this reason is extremely efficient for detecting not top lines, but extremely bad for detecting top lines (Sensitivity of 13.7% across traits and data sets), while the proposed reformulation method (model B) across traits and data sets is able to detect top lines with a probability of 66.4% (Sensitivity) and to detect not top lines with a probability of 62.2%, which by large margin better than the conventional regression model.

On the other hand, the second method, model RO, that is a postprocessing method outperform the conventional prediction model in terms of sensitivity by 402.9%, in terms of F1 score by 110.04% and in Kappa coefficient by 70.96% but resulted with worse efficacy in terms of specificity by 44.95%. These results illustrate that this proposed method guarantee a better sensitivity (larger than 0.5) and lower specificity (less than 0.7 and larger than 0.5) regarding the conventional method with sensitivity and specificity equal to 13.7% and 97.7% respectively. Also, in general terms produce similar or slightly better performance than the reformulation method (model B) that reformulate the regression model (model R) as a binary classification problem, but with the advantage that not required to reformulate the original regression problem of the GS methodology. Both methods outperform the conventional regression model (model R), but model RO, can be more attractive for its simplicity and good prediction performance.

In general, both methods (Model B and RO) offer a very flexible framework to improve the selection accuracy of the top lines. With regard to model B, it can be implemented with many conventional machine learning methods for classification like neural networks, gradient boosting machines, support vector machine, random forest, logistic regression, extreme gradient boosting machine, etc. However, to have a successful implementation of the proposed reformulation of the GS methodology, with model B, it is required to focus the training process to guarantee at least a similar sensitivity and specificity. However, also it is possible with the proposed reformulation method to perform a training process in such a way to obtain a significantly larger sensitivity and moderate specificity.

It is important to point out that both proposed methods (model B and RO) should be useful when the selection process is done regarding to checks or a threshold stablished regarding a quantile (80%, 90%, etc.) or any other mechanism. Because in these cases the sensitivity to detect the top lines is very low as was observed in the seven data sets used in this study (13.7%). For this reason, in these scenarios it is of paramount importance to adjust the thresholds, under both models for improving the sensitivity of selecting the top lines. In both proposed methods (model B and RO) their respective adjusted thresholds are shrunken to a lower value to guarantee at least a similar sensitivity and specificity. This shrinkage of the thresholds is required since in general machine learning models shrink the predicted values to the mean because they are not good in general to generalize out of the data used for training [12]. However, when the selection process will not be performed regarding to a threshold (performance of the best check, or average performance of checks, etc.) in the scale of the continues trait it is not required this adjustment for the thresholds since the breeder can select only a certain percentage of the top lines after making the ranking of the predicted genotypes.

The advantage of the proposed methods is that they guarantee that the classification of lines had a similar sensitivity and specificity since the classification process is performed in such a way that is minimized the average of the squared difference between the sensitivity and specificity. For this reason, in place of using the conventional thresholds (${Y}_{\tau }$ and $\tau$) for the classification of the predicted lines we used the optimal thresholds (${Y}_{\tau 0}$ and $\tau 0)$ that produces similar sensitivity and specificity since these optimal threshold were obtained by splitting each training set in inner-training and validation in such a way that with the validation set we can find these optimal thresholds that minimize the average of the squared difference between the sensitivity and specificity.

Regarding model RO, because we use the predictions resulting of using the conventional genomic prediction model, in this case the GBLUP model, the proposed method consists of only a postprocessing method that look for an optimal threshold (${Y}_{\tau 0})$for a better classification of the genotypes in the testing set. For this reason, this method even that produce similar or slightly better performance than model B, should be preferred since use the same machinery of conventional genomic prediction plus a simple postprocessing step.

Finally, it is important to point out that the proposed methods can be evaluated with other machine learning algorithms like random forest, gradient boosting machine, etc., to have a better picture of their performance. Also, variable selection methods can be incorporated to select only the optimal features to improve the efficiency of the proposed methods. Novel methods that had been applied for solving gene selection problem that combine existing metaheuristics like binary dragonfly algorithm (BDF) and binary black hole algorithm (BBHA) [31] can provide a significant increase in prediction accuracy if are combine with the two proposed methods in an appropriate way.

## Conclusions

In this research were proposed two methods to improve the accuracy of the conventional genomic selection methodology (formulated as a regression model). The first one reformulated the conventional regression model as a classification model, where top lines are labeled as ones and not top lines labeled as zeros according with a threshold that can be a quantile for the best (top lines) or the average (or maximum) performance of the checks. The second one is a simple postprocessing method to improve the sensitivity of detecting top lines under the conventional formulation of the genomic selection methodology. Our results shows that the two proposed models outperform by large margins the conventional genomic prediction models. For example across data sets in terms of F1 score, kappa coefficient and sensitivity the reformulation of the regression model as a classification model outperform the conventional prediction model (formulated as a regression problem) by 92.5, 33.5 and 386.6% respectively. While the simple postprocessing method outperforms the conventional regression model by 110.04, 70.96 and 402.9% in terms of F1 score, kappa coefficient and sensitivity, respectively. A successful implementation of the proposed models (Model B and RO) requires concentrating the learning process to guarantee a similar sensitivity and specificity. Also, in general the proposed postprocessing method produce equal or better prediction performance than the reformulation of the conventional genomic prediction methodology as a classification model. The advantage of the proposed simple postprocessing method over the reformulation as a classification method is its simplicity since only is required to modify the threshold to classify the continuous predictions resulting of the conventional prediction model. Given that our results are not conclusive we invite other scientist to evaluate with other data the proposed methods.

## Supplementary Information

**Additional file 1:** **Table A1.** Comparison between regression model, R, the classification model, B, and the regression optimum model, RO, for EYT_1 dataset in terms of F1 score, Kappa coefficient, Sensitivity and Specificity. SE denotes standard error, LL denotes lower limit and UL denotes upper limit and RE denotes relative efficiency. **Table A2.** Comparison between regression model, R, the classification model, B, and the regression optimum model, RO, for EYT_2 dataset in terms of F1 score, Kappa coefficient, Sensitivity and Specificity. SE denotes standard error, LL denotes lower limit and UL denotes upper limit and RE denotes relative efficiency. **Table A3.** Comparison between regression model, R, the classification model, B, and the regression optimum model, RO,  for Wheat_5 dataset in terms of F1 score, Kappa coefficient, Sensitivity and Specificity. SE denotes standard error, LL denotes lower limit and UL denotes upper limit and RE denotes relative efficiency. **Table A4.** Comparison between regression model, R, the classification model, B, and the regression optimum model, RO, for Wheat_6 dataset in terms of F1 score, Kappa coefficient, Sensitivity and Specificity. SE denotes standard error, LL denotes lower limit and UL denotes upper limit and RE denotes relative efficiency. **Table A5.** Comparison between regression model, R, the classification model, B, and the regression optimum model, RO,  for Across datasets in terms of F1score, Kappa coefficient, Sensitivity and Specificity. SE denotes standard error, LL denotes lower limit and UL denotes upper limit and RE denotes relative efficiency. **Table B1.** Comparison between regression model, R, the classification model, B, and the regression optimum model, RO, for EYT_3 dataset in terms of F1 score, Kappa coefficient, Sensitivity and Specificity. SE denotes Standard error, LL denotes lower limit and UL denotes upper limit and RE denotes relative efficiency. **Table B2.** Comparison between regression model, R, the classification model, B, and the regression optimum model, RO, for Wheat_1 dataset in terms of F1 score, Kappa coefficient, Sensitivity and Specificity. SE denotes Standard error, LL denotes lower limit and UL denotes upper limit and RE denotes relative efficiency. **Table B3.** Comparison between regression model, R, the classification model, B, and the regression optimum model, RO, for Wheat_4 dataset in terms of F1 score, Kappa coefficient, Sensitivity and Specificity. SE denotes Standard error, LL denotes lower limit and UL denotes upper limit and RE denotes relative efficiency. **Fig. S1.** A)Mean accuracy in terms of F1 score for classification model (B), regression model(R) and regression optimum model (RO) for trait GY for dataset EYT_3. B)Mean accuracy in terms of Kappa coefficient classification model (B), regression model (R) and regression optimum model (RO) for trait GY for dataset EYT_3. C)Mean accuracy in terms of sensitivity for classification model(B), regression model (R) and regression optimum model (RO) for trait GY for dataset EYT_3. D) Mean accuracy in terms of specificity for classification model (B), regression model (R) and regression optimum model (RO) for trait GY for dataset EYT_3. **Fig. S2.** A)Mean accuracy in terms of F1 score for classification model (B), regression model (R) and regression optimum model (RO) for trait GY for dataset Wheat_1. B)Mean accuracy in terms of Kappa coefficient for classification model (B), regression model (R) and regression optimum model (RO) for trait GY for dataset Wheat_1.C) Mean accuracy in terms of sensitivity for classification model (B),regression model (R) and regression optimum model (RO) for trait GY for dataset Wheat_1. D)Mean accuracy in terms of specificity for classification model(B), regression model (R) and regression optimum model (RO) for trait GY for dataset Wheat_1. **Fig. S3.** A)Mean accuracy in terms of F1 score for classification model (B), regression model (R) and regression optimum model (RO) for trait GY for dataset Wheat_4. B) Mean accuracy in terms of Kappa coefficient for classification model (B), regression model (R) and regression optimum model (RO) for trait GY for dataset Wheat_4. C)Mean accuracy in terms of sensitivity for classification model (B), regression model (R) and regression optimum model (RO) for trait GY for dataset Wheat_4. D) Mean accuracy in terms of specificity for classification model (B), regression model (R) and regression optimum model (RO) for trait GY for dataset Wheat_4.

## Data Availability

The phenotypic and genomic information for each data set employed on this study can be downloaded from the links: https://data.cimmyt.org/dataset.xhtml?persistentId=hdl:11529/10548423 and https://data.cimmyt.org/dataset.xhtml?persistentId=hdl:11529/10548140.
