# Supplementary material for: Two simple methods to improve the accuracy of the genomic selection methodology
Source: BMC Genomics. 2023 Apr 26;24:220. doi: 10.1186/s12864-023-09294-5 (PMC10131336; doi:10.1186/s12864-023-09294-5)
Supplement: Supplementary file 1 — Additional file 1: Table A1. Comparison between regression model, R, the classification model, B, and the regression optimum model, RO, for EYT_1 dataset in terms of F1 score, Kappa coefficient, Sensitivity and Specificity. SE denotes standard error, LL denotes lower limit and UL denotes upper limit and RE denotes relative efficiency. Table A2. Comparison between regression model, R, the classification model, B, and the regression optimum model, RO, for EYT_2 dataset in terms of F1 score, Kappa coefficient, Sensitivity and Specificity. SE denotes standard error, LL denotes lower limit and UL denotes upper limit and RE denotes relative efficiency. Table A3. Comparison between regression model, R, the classification model, B, and the regression optimum model, RO, for Wheat_5 dataset in terms of F1 score, Kappa coefficient, Sensitivity and Specificity. SE denotes standard error, LL denotes lower limit and UL denotes upper limit and RE denotes relative efficiency. Table A4. Comparison between regression model, R, the classification model, B, and the regression optimum model, RO, for Wheat_6 dataset in terms of F1 score, Kappa coefficient, Sensitivity and Specificity. SE denotes standard error, LL denotes lower limit and UL denotes upper limit and RE denotes relative efficiency. Table A5. Comparison between regression model, R, the classification model, B, and the regression optimum model, RO, for Across datasets in terms of F1score, Kappa coefficient, Sensitivity and Specificity. SE denotes standard error, LL denotes lower limit and UL denotes upper limit and RE denotes relative efficiency. Table B1. Comparison between regression model, R, the classification model, B, and the regression optimum model, RO, for EYT_3 dataset in terms of F1 score, Kappa coefficient, Sensitivity and Specificity. SE denotes Standard error, LL denotes lower limit and UL denotes upper limit and RE denotes relative efficiency. Table B2. Comparison between regression model, R, the classification [file 12864_2023_9294_MOESM1_ESM.docx]

# Appendix A

## Table A1. Comparison between regression model, R, the classification model, B, and the regression optimum model, RO, for EYT_1 dataset in terms of F1 score, Kappa coefficient, Sensitivity and Specificity. SE denotes standard error, LL denotes lower limit and UL denotes upper limit and RE denotes relative efficiency.

| **Metrics** | **Model** | **Trait** | **Mean** | **SE** | **UL** | **LL** | **RE** |
| --- | --- | --- | --- | --- | --- | --- | --- |
| F1 | B | DTHD | 0.357 | 0.030 | 0.387 | 0.326 |  |
| F1 | B | DTMT | 0.345 | 0.031 | 0.376 | 0.314 |  |
| F1 | B | GY | 0.411 | 0.017 | 0.428 | 0.394 |  |
| F1 | B | Height | 0.383 | 0.026 | 0.409 | 0.356 |  |
| Kappa | B | DTHD | 0.109 | 0.036 | 0.145 | 0.073 |  |
| Kappa | B | DTMT | 0.092 | 0.023 | 0.114 | 0.069 |  |
| Kappa | B | GY | 0.180 | 0.015 | 0.195 | 0.165 |  |
| Kappa | B | Height | 0.140 | 0.024 | 0.164 | 0.116 |  |
| Sensitivity | B | DTHD | 0.633 | 0.080 | 0.713 | 0.553 |  |
| Sensitivity | B | DTMT | 0.593 | 0.029 | 0.622 | 0.564 |  |
| Sensitivity | B | GY | 0.696 | 0.026 | 0.722 | 0.670 |  |
| Sensitivity | B | Height | 0.663 | 0.035 | 0.698 | 0.628 |  |
| Specificity | B | DTHD | 0.548 | 0.034 | 0.581 | 0.514 |  |
| Specificity | B | DTMT | 0.544 | 0.021 | 0.565 | 0.523 |  |
| Specificity | B | GY | 0.577 | 0.020 | 0.597 | 0.558 |  |
| Specificity | B | Height | 0.551 | 0.025 | 0.576 | 0.526 |  |
| F1 | R | DTHD | - | - | - | - | - |
| F1 | R | DTMT | 0.103 | 0.033 | 0.136 | 0.070 | 3.350 |
| F1 | R | GY | 0.215 | 0.046 | 0.260 | 0.169 | 1.913 |
| F1 | R | Height | 0.226 | 0.017 | 0.243 | 0.209 | 1.693 |
| Kappa | R | DTHD | 0.070 | 0.037 | 0.107 | 0.033 | 1.559 |
| Kappa | R | DTMT | 0.046 | 0.028 | 0.074 | 0.018 | 1.986 |
| Kappa | R | GY | 0.164 | 0.044 | 0.208 | 0.120 | 1.099 |
| Kappa | R | Height | 0.169 | 0.016 | 0.185 | 0.153 | 0.828 |
| Sensitivity | R | DTHD | 0.076 | 0.032 | 0.108 | 0.044 | 8.348 |
| Sensitivity | R | DTMT | 0.063 | 0.022 | 0.086 | 0.041 | 9.336 |
| Sensitivity | R | GY | 0.128 | 0.028 | 0.156 | 0.100 | 5.442 |
| Sensitivity | R | Height | 0.137 | 0.011 | 0.149 | 0.126 | 4.832 |
| Specificity | R | DTHD | 0.976 | 0.006 | 0.981 | 0.970 | 0.562 |
| Specificity | R | DTMT | 0.970 | 0.009 | 0.979 | 0.960 | 0.561 |
| Specificity | R | GY | 0.987 | 0.004 | 0.991 | 0.983 | 0.585 |
| Specificity | R | Height | 0.982 | 0.004 | 0.986 | 0.978 | 0.561 |
| F1 | RO | DTHD | 0.408 | 0.023 | 0.431 | 0.386 | 0.874 |
| F1 | RO | DTMT | 0.391 | 0.012 | 0.403 | 0.380 | 0.881 |
| F1 | RO | GY | 0.487 | 0.021 | 0.508 | 0.465 | 0.844 |
| F1 | RO | Height | 0.415 | 0.015 | 0.430 | 0.400 | 0.921 |
| Kappa | RO | DTHD | 0.190 | 0.021 | 0.211 | 0.169 | 0.574 |
| Kappa | RO | DTMT | 0.166 | 0.012 | 0.178 | 0.154 | 0.552 |
| Kappa | RO | GY | 0.304 | 0.026 | 0.331 | 0.278 | 0.592 |
| Kappa | RO | Height | 0.200 | 0.026 | 0.227 | 0.174 | 0.699 |
| Sensitivity | RO | DTHD | 0.654 | 0.023 | 0.677 | 0.630 | 0.968 |
| Sensitivity | RO | DTMT | 0.634 | 0.042 | 0.675 | 0.592 | 0.936 |
| Sensitivity | RO | GY | 0.711 | 0.043 | 0.753 | 0.668 | 0.979 |
| Sensitivity | RO | Height | 0.639 | 0.040 | 0.679 | 0.599 | 1.038 |
| Specificity | RO | DTHD | 0.621 | 0.025 | 0.646 | 0.596 | 0.882 |
| Specificity | RO | DTMT | 0.614 | 0.024 | 0.638 | 0.590 | 0.886 |
| Specificity | RO | GY | 0.699 | 0.019 | 0.718 | 0.681 | 0.825 |
| Specificity | RO | Height | 0.647 | 0.017 | 0.663 | 0.630 | 0.852 |

## Table A2. Comparison between regression model, R, the classification model, B, and the regression optimum model, RO, for EYT_2 dataset in terms of F1 score, Kappa coefficient, Sensitivity and Specificity. SE denotes standard error, LL denotes lower limit and UL denotes upper limit and RE denotes relative efficiency.

| **Metrics** | **Model** | **Trait** | **Mean** | **SE** | **UL** | **LL** | **RE** |
| --- | --- | --- | --- | --- | --- | --- | --- |
| F1 | B | DTHD | 0.387 | 0.015 | 0.402 | 0.372 |  |
| F1 | B | DTMT | 0.411 | 0.019 | 0.430 | 0.393 |  |
| F1 | B | GY | 0.418 | 0.014 | 0.432 | 0.404 |  |
| F1 | B | Height | 0.354 | 0.041 | 0.395 | 0.312 |  |
| Kappa | B | DTHD | 0.135 | 0.033 | 0.168 | 0.102 |  |
| Kappa | B | DTMT | 0.175 | 0.021 | 0.196 | 0.154 |  |
| Kappa | B | GY | 0.183 | 0.011 | 0.194 | 0.172 |  |
| Kappa | B | Height | 0.163 | 0.041 | 0.204 | 0.122 |  |
| Sensitivity | B | DTHD | 0.705 | 0.016 | 0.721 | 0.689 |  |
| Sensitivity | B | DTMT | 0.729 | 0.037 | 0.767 | 0.692 |  |
| Sensitivity | B | GY | 0.748 | 0.010 | 0.758 | 0.738 |  |
| Sensitivity | B | Height | 0.551 | 0.110 | 0.661 | 0.440 |  |
| Specificity | B | DTHD | 0.509 | 0.046 | 0.555 | 0.463 |  |
| Specificity | B | DTMT | 0.549 | 0.023 | 0.573 | 0.526 |  |
| Specificity | B | GY | 0.543 | 0.007 | 0.550 | 0.537 |  |
| Specificity | B | Height | 0.665 | 0.091 | 0.757 | 0.574 |  |
| F1 | R | DTHD | 0.209 | 0.028 | 0.237 | 0.181 | 1.850 |
| F1 | R | DTMT | 0.361 | 0.037 | 0.398 | 0.324 | 1.140 |
| F1 | R | GY | 0.343 | 0.030 | 0.374 | 0.313 | 1.217 |
| F1 | R | Height | 0.283 | 0.022 | 0.305 | 0.261 | 1.249 |
| Kappa | R | DTHD | 0.142 | 0.019 | 0.161 | 0.123 | 0.949 |
| Kappa | R | DTMT | 0.292 | 0.031 | 0.323 | 0.261 | 0.599 |
| Kappa | R | GY | 0.259 | 0.034 | 0.293 | 0.225 | 0.707 |
| Kappa | R | Height | 0.222 | 0.026 | 0.248 | 0.197 | 0.732 |
| Sensitivity | R | DTHD | 0.133 | 0.021 | 0.154 | 0.111 | 5.314 |
| Sensitivity | R | DTMT | 0.248 | 0.035 | 0.283 | 0.213 | 2.942 |
| Sensitivity | R | GY | 0.242 | 0.024 | 0.266 | 0.218 | 3.094 |
| Sensitivity | R | Height | 0.178 | 0.016 | 0.194 | 0.162 | 3.095 |
| Specificity | R | DTHD | 0.971 | 0.006 | 0.977 | 0.965 | 0.524 |
| Specificity | R | DTMT | 0.976 | 0.007 | 0.983 | 0.969 | 0.563 |
| Specificity | R | GY | 0.960 | 0.005 | 0.965 | 0.954 | 0.566 |
| Specificity | R | Height | 0.982 | 0.005 | 0.987 | 0.977 | 0.677 |
| F1 | RO | DTHD | 0.412 | 0.027 | 0.439 | 0.385 | 0.939 |
| F1 | RO | DTMT | 0.479 | 0.035 | 0.514 | 0.444 | 0.858 |
| F1 | RO | GY | 0.508 | 0.040 | 0.548 | 0.468 | 0.822 |
| F1 | RO | Height | 0.467 | 0.021 | 0.488 | 0.445 | 0.758 |
| Kappa | RO | DTHD | 0.195 | 0.037 | 0.232 | 0.159 | 0.692 |
| Kappa | RO | DTMT | 0.292 | 0.042 | 0.334 | 0.250 | 0.600 |
| Kappa | RO | GY | 0.337 | 0.052 | 0.388 | 0.285 | 0.544 |
| Kappa | RO | Height | 0.281 | 0.020 | 0.301 | 0.261 | 0.580 |
| Sensitivity | RO | DTHD | 0.642 | 0.055 | 0.697 | 0.587 | 1.098 |
| Sensitivity | RO | DTMT | 0.713 | 0.045 | 0.758 | 0.668 | 1.023 |
| Sensitivity | RO | GY | 0.718 | 0.048 | 0.766 | 0.670 | 1.043 |
| Sensitivity | RO | Height | 0.672 | 0.022 | 0.694 | 0.650 | 0.819 |
| Specificity | RO | DTHD | 0.637 | 0.020 | 0.657 | 0.617 | 0.799 |
| Specificity | RO | DTMT | 0.685 | 0.023 | 0.708 | 0.662 | 0.802 |
| Specificity | RO | GY | 0.720 | 0.026 | 0.746 | 0.694 | 0.754 |
| Specificity | RO | Height | 0.704 | 0.018 | 0.722 | 0.686 | 0.946 |

## Table A3. Comparison between regression model, R, the classification model, B, and the regression optimum model, RO, for Wheat_5 dataset in terms of F1 score, Kappa coefficient, Sensitivity and Specificity. SE denotes standard error, LL denotes lower limit and UL denotes upper limit and RE denotes relative efficiency.

| **Metrics** | **Model** | **Trait** | **Mean** | **SE** | **UL** | **LL** | **RE** |
| --- | --- | --- | --- | --- | --- | --- | --- |
| F1 | B | GY | 0.454 | 0.019 | 0.473 | 0.434 |  |
| Kappa | B | GY | 0.256 | 0.022 | 0.278 | 0.234 |  |
| Sensitivity | B | GY | 0.677 | 0.014 | 0.692 | 0.663 |  |
| Specificity | B | GY | 0.673 | 0.017 | 0.691 | 0.656 |  |
| F1 | R | GY | 0.174 | 0.033 | 0.207 | 0.141 | 2.614 |
| Kappa | R | GY | 0.123 | 0.030 | 0.153 | 0.093 | 2.082 |
| Sensitivity | R | GY | 0.105 | 0.022 | 0.127 | 0.082 | 6.464 |
| Specificity | R | GY | 0.981 | 0.004 | 0.986 | 0.977 | 0.686 |
| F1 | RO | GY | 0.467 | 0.025 | 0.492 | 0.441 | 0.973 |
| Kappa | RO | GY | 0.275 | 0.025 | 0.300 | 0.249 | 0.932 |
| Sensitivity | RO | GY | 0.698 | 0.036 | 0.733 | 0.662 | 0.971 |
| Specificity | RO | GY | 0.679 | 0.017 | 0.696 | 0.661 | 0.992 |

## Table A4. Comparison between regression model, R, the classification model, B, and the regression optimum model, RO, for Wheat_6 dataset in terms of F1 score, Kappa coefficient, Sensitivity and Specificity. SE denotes standard error, LL denotes lower limit and UL denotes upper limit and RE denotes relative efficiency.

| **Metrics** | **Model** | **Trait** | **Mean** | **SE** | **UL** | **LL** | **RE** |
| --- | --- | --- | --- | --- | --- | --- | --- |
| F1 | B | GY | 0.505 | 0.014 | 0.520 | 0.491 |  |
| Kappa | B | GY | 0.333 | 0.015 | 0.349 | 0.318 |  |
| Sensitivity | B | GY | 0.713 | 0.023 | 0.736 | 0.690 |  |
| Specificity | B | GY | 0.725 | 0.009 | 0.734 | 0.716 |  |
| F1 | R | GY | 0.305 | 0.041 | 0.346 | 0.263 | 1.658 |
| Kappa | R | GY | 0.225 | 0.040 | 0.265 | 0.185 | 1.481 |
| Sensitivity | R | GY | 0.207 | 0.032 | 0.239 | 0.176 | 3.441 |
| Specificity | R | GY | 0.965 | 0.004 | 0.969 | 0.960 | 0.752 |
| F1 | RO | GY | 0.516 | 0.017 | 0.532 | 0.499 | 0.979 |
| Kappa | RO | GY | 0.347 | 0.017 | 0.363 | 0.330 | 0.962 |
| Sensitivity | RO | GY | 0.737 | 0.027 | 0.764 | 0.709 | 0.968 |
| Specificity | RO | GY | 0.723 | 0.007 | 0.730 | 0.717 | 1.003 |

## Table A5. Comparison between regression model, R, the classification model, B, and the regression optimum model, RO, for Across datasets in terms of F1 score, Kappa coefficient, Sensitivity and Specificity. SE denotes standard error, LL denotes lower limit and UL denotes upper limit and RE denotes relative efficiency.

| **Metrics** | **Model** | **Mean** | **SE** | **UL** | **LL** | **RE** |
| --- | --- | --- | --- | --- | --- | --- |
| F1 | B | 0.421 | 0.022 | 0.443 | 0.398 |  |
| Kappa | B | 0.207 | 0.024 | 0.232 | 0.183 |  |
| Sensitivity | B | 0.664 | 0.035 | 0.699 | 0.629 |  |
| Specificity | B | 0.626 | 0.026 | 0.652 | 0.600 |  |
| F1 | R | 0.219 | 0.033 | 0.251 | 0.186 | 1.925 |
| Kappa | R | 0.155 | 0.031 | 0.187 | 0.124 | 1.335 |
| Sensitivity | R | 0.137 | 0.024 | 0.160 | 0.113 | 4.866 |
| Specificity | R | 0.977 | 0.005 | 0.983 | 0.972 | 0.640 |
| F1 | RO | 0.460 | 0.025 | 0.485 | 0.435 | 0.915 |
| Kappa | RO | 0.265 | 0.029 | 0.294 | 0.236 | 0.781 |
| Sensitivity | RO | 0.689 | 0.037 | 0.726 | 0.652 | 0.964 |
| Specificity | RO | 0.674 | 0.016 | 0.691 | 0.658 | 0.928 |

**Appendix B**

## Table B1. Comparison between regression model, R, the classification model, B, and the regression optimum model, RO, for EYT_3 dataset in terms of F1 score, Kappa coefficient, Sensitivity and Specificity. SE denotes Standard error, LL denotes lower limit and UL denotes upper limit and RE denotes relative efficiency.

| **Metrics** | **Model** | **Trait** | **Mean** | **SE** | **UL** | **LL** | **RE** |
| --- | --- | --- | --- | --- | --- | --- | --- |
| F1 | B | DTHD | 0.330 | 0.018 | 0.348 | 0.312 |  |
| F1 | B | DTMT | 0.387 | 0.031 | 0.418 | 0.355 |  |
| F1 | B | GY | 0.444 | 0.016 | 0.460 | 0.428 |  |
| F1 | B | Height | 0.417 | 0.014 | 0.431 | 0.403 |  |
| Kappa | B | DTHD | 0.062 | 0.025 | 0.088 | 0.037 |  |
| Kappa | B | DTMT | 0.126 | 0.020 | 0.147 | 0.106 |  |
| Kappa | B | GY | 0.223 | 0.012 | 0.235 | 0.211 |  |
| Kappa | B | Height | 0.173 | 0.004 | 0.178 | 0.169 |  |
| Sensitivity | B | DTHD | 0.651 | 0.090 | 0.742 | 0.561 |  |
| Sensitivity | B | DTMT | 0.783 | 0.023 | 0.806 | 0.760 |  |
| Sensitivity | B | GY | 0.777 | 0.021 | 0.798 | 0.756 |  |
| Sensitivity | B | Height | 0.801 | 0.013 | 0.814 | 0.787 |  |
| Specificity | B | DTHD | 0.471 | 0.049 | 0.519 | 0.422 |  |
| Specificity | B | DTMT | 0.437 | 0.003 | 0.441 | 0.434 |  |
| Specificity | B | GY | 0.572 | 0.020 | 0.592 | 0.552 |  |
| Specificity | B | Height | 0.495 | 0.013 | 0.508 | 0.482 |  |
| F1 | R | DTHD | - | - | - | - | - |
| F1 | R | DTMT | 0.214 | 0.038 | 0.252 | 0.176 | 1.809 |
| F1 | R | GY | 0.293 | 0.052 | 0.344 | 0.241 | 1.517 |
| F1 | R | Height | 0.268 | 0.048 | 0.316 | 0.220 | 1.555 |
| Kappa | R | DTHD | 0.114 | 0.048 | 0.162 | 0.065 | 0.545 |
| Kappa | R | DTMT | 0.163 | 0.037 | 0.200 | 0.127 | 0.773 |
| Kappa | R | GY | 0.225 | 0.042 | 0.267 | 0.182 | 0.992 |
| Kappa | R | Height | 0.195 | 0.046 | 0.241 | 0.149 | 0.888 |
| Sensitivity | R | DTHD | 0.096 | 0.035 | 0.131 | 0.061 | 6.776 |
| Sensitivity | R | DTMT | 0.128 | 0.025 | 0.153 | 0.102 | 6.138 |
| Sensitivity | R | GY | 0.196 | 0.039 | 0.234 | 0.157 | 3.969 |
| Sensitivity | R | Height | 0.180 | 0.037 | 0.217 | 0.143 | 4.440 |
| Specificity | R | DTHD | 0.984 | 0.005 | 0.989 | 0.980 | 0.478 |
| Specificity | R | DTMT | 0.987 | 0.003 | 0.991 | 0.984 | 0.443 |
| Specificity | R | GY | 0.974 | 0.006 | 0.980 | 0.968 | 0.588 |
| Specificity | R | Height | 0.968 | 0.004 | 0.971 | 0.964 | 0.512 |
| F1 | RO | DTHD | 0.448 | 0.015 | 0.462 | 0.433 | 0.737 |
| F1 | RO | DTMT | 0.497 | 0.036 | 0.533 | 0.461 | 0.778 |
| F1 | RO | GY | 0.488 | 0.020 | 0.509 | 0.468 | 0.910 |
| F1 | RO | Height | 0.489 | 0.029 | 0.518 | 0.460 | 0.853 |
| Kappa | RO | DTHD | 0.245 | 0.026 | 0.271 | 0.220 | 0.253 |
| Kappa | RO | DTMT | 0.322 | 0.040 | 0.362 | 0.282 | 0.393 |
| Kappa | RO | GY | 0.310 | 0.016 | 0.326 | 0.293 | 0.720 |
| Kappa | RO | Height | 0.303 | 0.042 | 0.345 | 0.261 | 0.572 |
| Sensitivity | RO | DTHD | 0.692 | 0.042 | 0.734 | 0.649 | 0.941 |
| Sensitivity | RO | DTMT | 0.726 | 0.022 | 0.748 | 0.705 | 1.078 |
| Sensitivity | RO | GY | 0.711 | 0.042 | 0.753 | 0.669 | 1.093 |
| Sensitivity | RO | Height | 0.734 | 0.046 | 0.781 | 0.688 | 1.090 |
| Specificity | RO | DTHD | 0.661 | 0.010 | 0.671 | 0.651 | 0.712 |
| Specificity | RO | DTMT | 0.705 | 0.021 | 0.726 | 0.684 | 0.620 |
| Specificity | RO | GY | 0.705 | 0.020 | 0.725 | 0.685 | 0.811 |
| Specificity | RO | Height | 0.686 | 0.021 | 0.707 | 0.664 | 0.722 |

## Table B2. Comparison between regression model, R, the classification model, B, and the regression optimum model, RO, for Wheat_1 dataset in terms of F1 score, Kappa coefficient, Sensitivity and Specificity. SE denotes Standard error, LL denotes lower limit and UL denotes upper limit and RE denotes relative efficiency.

| **Metrics** | **Model** | **Trait** | **Mean** | **SE** | **UL** | **LL** | **RE** |
| --- | --- | --- | --- | --- | --- | --- | --- |
| F1 | B | GY | 0.411 | 0.033 | 0.444 | 0.378 |  |
| Kappa | B | GY | 0.201 | 0.033 | 0.234 | 0.168 |  |
| Sensitivity | B | GY | 0.615 | 0.020 | 0.635 | 0.595 |  |
| Specificity | B | GY | 0.657 | 0.016 | 0.674 | 0.641 |  |
| F1 | R | GY | 0.244 | 0.036 | 0.279 | 0.208 | 1.686 |
| Kappa | R | GY | 0.174 | 0.033 | 0.207 | 0.140 | 1.157 |
| Sensitivity | R | GY | 0.156 | 0.023 | 0.180 | 0.133 | 3.938 |
| Specificity | R | GY | 0.971 | 0.009 | 0.980 | 0.962 | 0.677 |
| F1 | RO | GY | 0.417 | 0.041 | 0.458 | 0.376 | 0.986 |
| Kappa | RO | GY | 0.204 | 0.043 | 0.247 | 0.162 | 0.983 |
| Sensitivity | RO | GY | 0.647 | 0.037 | 0.684 | 0.609 | 0.951 |
| Specificity | RO | GY | 0.638 | 0.014 | 0.652 | 0.623 | 1.031 |

## Table B3. Comparison between regression model, R, the classification model, B, and the regression optimum model, RO, for Wheat_4 dataset in terms of F1 score, Kappa coefficient, Sensitivity and Specificity. SE denotes Standard error, LL denotes lower limit and UL denotes upper limit and RE denotes relative efficiency.

| **Metrics** | **Model** | **Trait** | **Mean** | **SE** | **UL** | **LL** | **RE** |
| --- | --- | --- | --- | --- | --- | --- | --- |
| Kappa | B | GY | 0.220 | 0.035 | 0.255 | 0.185 |  |
| Sensitivity | B | GY | 0.562 | 0.065 | 0.627 | 0.497 |  |
| Specificity | B | GY | 0.709 | 0.052 | 0.761 | 0.657 |  |
| F1 | B | GY | 0.414 | 0.022 | 0.436 | 0.392 |  |
| Kappa | R | GY | 0.049 | 0.014 | 0.064 | 0.035 | 4.445 |
| Sensitivity | R | GY | 0.036 | 0.006 | 0.042 | 0.030 | 15.520 |
| Specificity | R | GY | 0.995 | 0.004 | 0.999 | 0.992 | 0.713 |
| F1 | R | GY | 0.069 | 0.011 | 0.080 | 0.058 | 6.001 |
| Kappa | RO | GY | 0.244 | 0.027 | 0.271 | 0.218 | 0.900 |
| Sensitivity | RO | GY | 0.680 | 0.042 | 0.722 | 0.639 | 0.826 |
| Specificity | RO | GY | 0.660 | 0.016 | 0.676 | 0.644 | 1.074 |
| F1 | RO | GY | 0.447 | 0.020 | 0.467 | 0.427 | 0.926 |

**Supplementary material**

# EYT_3

As can be seen in Fig. S1 the proposed reformulation model, B, has better prediction performance than the convention regression model, R, but not has better prediction performance than the regression optimum model, RO. In terms of F1 score the these were the performance in traits DTHD (B=0.330, R=NA, RO=0.448), DTMT (B=0.387, R=0.214, RO=0.497), GY (B=0.444, R=0.293, RO=0.488) and Height (B=0.417, R=0.268, RO=0.489), that is, in terms of F1 score model B outperformed model R in trait DTMT by 80.9% (RE=1.809), in trait GY by 51.7% (RE=1.517) and in trait Height by 55.5% (RE=1.555). Also, in terms of F1 score model RO outperformed model R in trait DTHD by 26.3%, in trait DTMT by 22.2%, in trait GY by 9.0% and in trait Height by 14.7%, for details see appendix Table B1 and Fig. S1A. Also, in terms of Kappa coefficient, models R and RO have better prediction performance than the classification model, B, in traits DTHD (B=0.062, R=0.114, RO=0.245), DTMT (B=0.126, R=0.163, RO=0.322), GY (B=0.223, R=0.225, RO=0.310) and Height (B=0.173, R=0.195, RO=0.303), in other words in terms of Kappa coefficient model R outperformed model B by 45.5% for trait DTHD, 22.7% for trait DTMT, 0.8% for trait GY and 11.2% for trait Height. Also, in terms of Kappa coefficient model RO outperformed model B by 74.7% in trait DTHD, by 60.7% in trait DTMT, by 28.0% in trait GY and by 42.8% in trait Height, for details see in appendix Table B1 and Fig. S1B. Also, in terms of sensitivity the classification modelwas better than the R and RO models in some traits, in traits DTHD (B=0.651, R=0.096, RO=0.692), DTMT (B=0.783, R=0.128, RO=0.726), GY (B=0.777, R=0.196, RO=0.711) and Height (B=0.801, R=0.180, RO=0.734), that is, in terms of sensitivity model B outperformed model R by 577.6% (RE=6.776), 513.8% (RE=6.138), 296.9% (RE=3.969) and 344.0% (RE=4.440%) for traits DTHD, DTMT, GY and Height respectively. Also, in terms of sensitivity model B outperformed model RO by 7.8% (RE=1.078 in trait DTHD), by 9.3% (RE=1.093 in trait GY) and by 9.0% (RE=1.090 in trait Height) while also in terms of sensitivity model RO outperformed model B by 5.9% in trait DTHD, see details in appendix Table B1 and Fig. S1C. Finally, in terms of specificity the R and RO models showed better performance in the four traits DTHD (B=0.471, R=0.984, RO=0.661), DTMT (B=0.437, R=0.987, RO=0.705), GY (B=0.572, R=0.974, RO=0.705) and Height (B=0.495, R=0.968, RO=0.686), that is, model R outperformed model B by 52.2% in trait DTHD, by 55.7% in trait DTMT, by 41.2% in trait GY and by 48.8% in trait Height. Also, in terms of specificity model RO outperformed model B by 28.8% in trait DTHD, by 38.0% in trait DTMT, by 18.9% in trait GY and by 27.8% in trait Height for details see in appendix Table B1 and Fig. S1D.


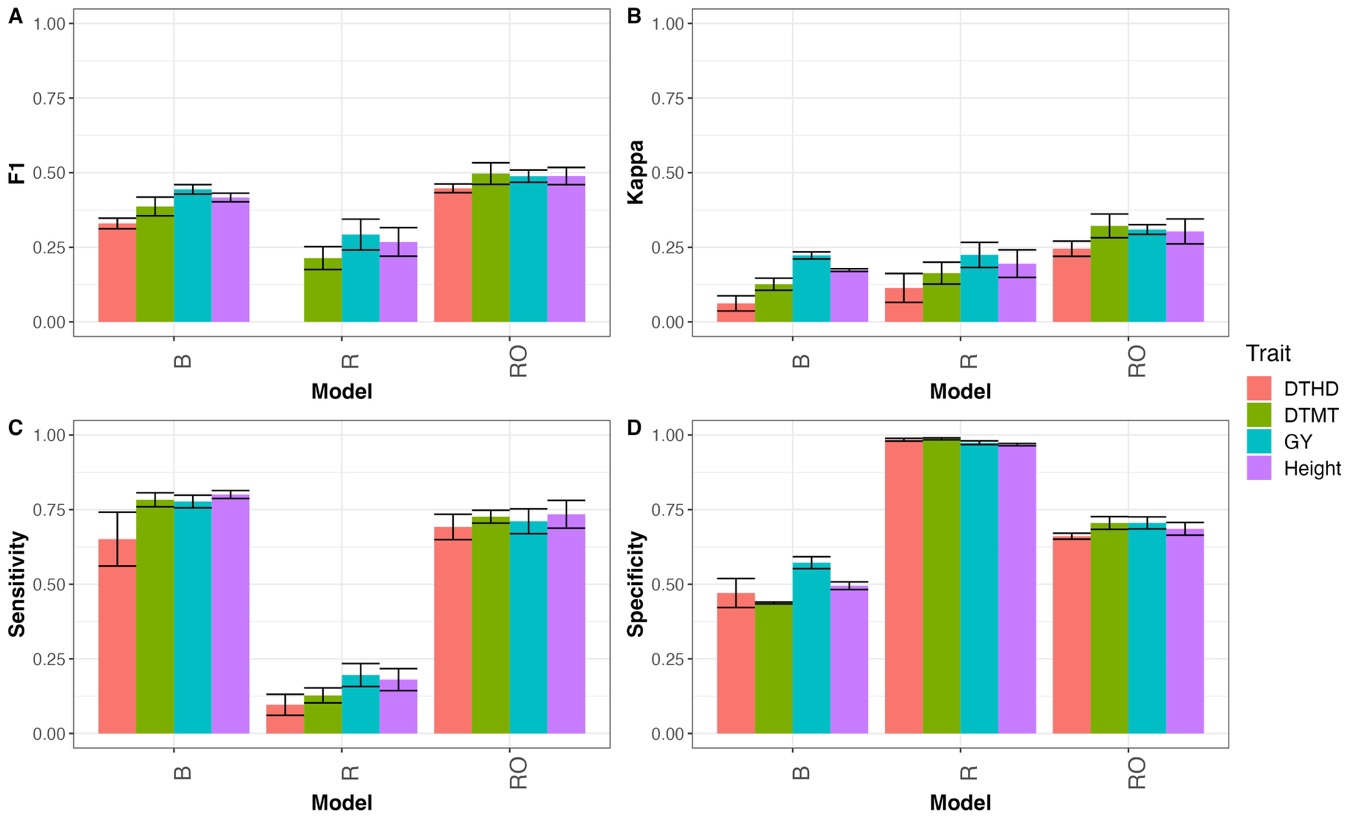


**Fig. S1 A)** Mean accuracy in terms of F1 score for classification model (B), regression model (R) and regression optimum model (RO) for trait GY for dataset EYT_3. **B)** Mean accuracy in terms of Kappa coefficient classification model(B), regression model (R) and regression optimum model (RO) for trait GY for dataset EYT_3. **C)** Mean accuracy in terms of sensibility for classification model(B), regression model (R) and regression optimum model (RO) for trait GY for dataset EYT_3. **D)** Mean accuracy in terms of specificity for classification model(B), regression model (R) and regression optimum model (RO) for trait GY for dataset EYT_3.

# Wheat_1

As can be seen in Fig. S2 the proposed model, RO, has better prediction performance than the convention regression model, R and the reformulation model B. In terms of F1 score this was the performance in trait GY (B = 0.411, R=0.244, RO=0.417), that is, in terms of F1 score model B outperformed model R in trait GY by 68.6% (RE=1.686), while model RO outperformed model B in trait GY by 1.4%, for details see appendix Table B2 and Fig. S2A. Also, in terms of Kappa coefficient, the Classification model outperformed the regression model but not the RO model in trait GY (B=0.201, R=0.174, RO=0.204), in other words in terms of Kappa coefficient model B outperformed model R by 15.7% (RE=1.157 for trait GY), and model RO outperformed model B by 1.7% for trait GY, for details see in appendix Table B2 and Fig. S2B. Also, in terms of sensitivity the classification model was better than the regression model, but not than model RO in GY trait (B=0.615, R=0.156, RO=0.647), that is, in terms of sensitivity model B outperformed model R by 293.8% (RE=3.938), and model RO outperformed model B by 4.9% in GY trait, for details see in appendix Table B2 and Fig. S2C. Finally, in terms of specificity the regression model and regression optimum model showed better performance than model B model in GY trait (B=0.657, R=0.971, RO=0.638), that is, model R outperformed model B, by 32.3% in trait GY and model RO by 3.1% in trait GY, for details see in appendix Table B2 and Fig. S2D.


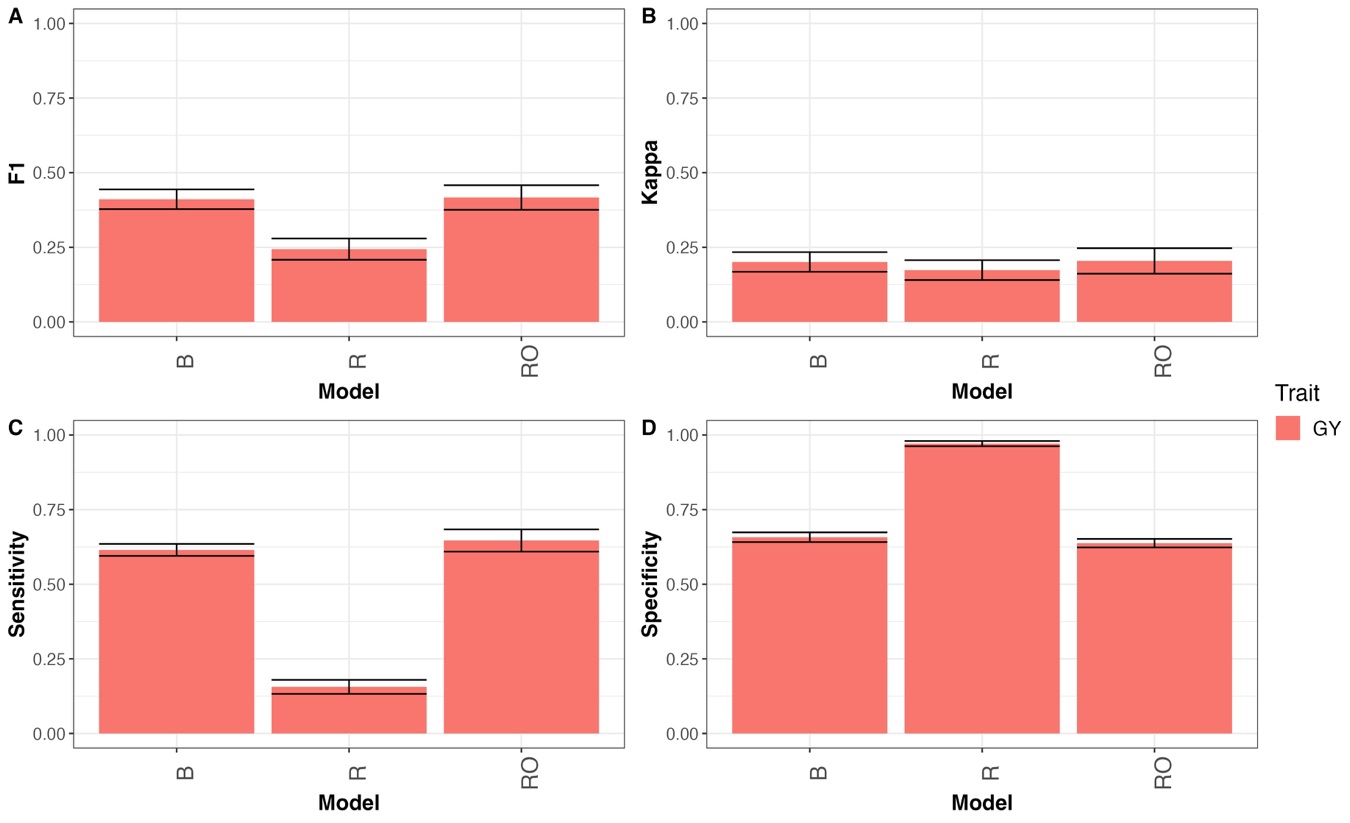


**Fig. S2 A)** Mean accuracy in terms of F1 score for classification model (B), regression model (R) and regression optimum model (RO) for trait GY for dataset Wheat_1. **B)** Mean accuracy in terms of Kappa coefficient for classification model(B), regression model (R) and regression optimum model (RO) for trait GY for dataset Wheat_1. **C)** Mean accuracy in terms of sensibility for classification model(B), regression model (R) and regression optimum model (RO) for trait GY for dataset Wheat_1. **D)** Mean accuracy in terms of specificity for classification model(B), regression model (R) and regression optimum model (RO) for trait GY for dataset Wheat_1.

# Wheat_4

As can be seen in Fig. S3 model B, has better prediction performance than the conventional regression model, R, but not has better prediction performance than the regression optimum model, RO. In terms of F1 score this was the performance in trait GY (B = 0.414, R=0.069, RO=0.447), that is, in terms of F1 score model B outperformed model R in trait GY by 500.1% (RE=6.001), in terms of F1 score model RO outperformed model B in trait GY by 7.4% for details see appendix Table B3 and Fig. S3A. Also, in terms of Kappa coefficient, the Classification model outperformed the regression model but not the RO model in trait GY (B=0.220, R=0.049, RO=0.244), in other words in terms of Kappa coefficient model B outperformed model R by 344.5% (RE=4.445 for trait GY), and model RO outperformed model B by 10.0% for trait GY, for details see in appendix Table B3 and Fig. S3B. Also, in terms of sensitivity the classification model was better than the regression model, but not for RO model in GY trait (B=0.562, R=0.036, RO=0.680), that is, in terms of Sensitivity model B outperformed model R by 1452.0% (RE=15.520), and model RO outperformed model B by 17.4% in GY trait, for details see in appendix Table B3 and Fig. S3C. Finally, in terms of specificity the regression model and regression optimum model showed better performance than B model in GY trait (B=0.709, R=0.995, RO=0.660), that is model R outperformed model B, by 28.7% in trait GY and the B model outperformed model RO by 7.4% in trait GY, for details see in appendix Table B3 and Fig. S3D.


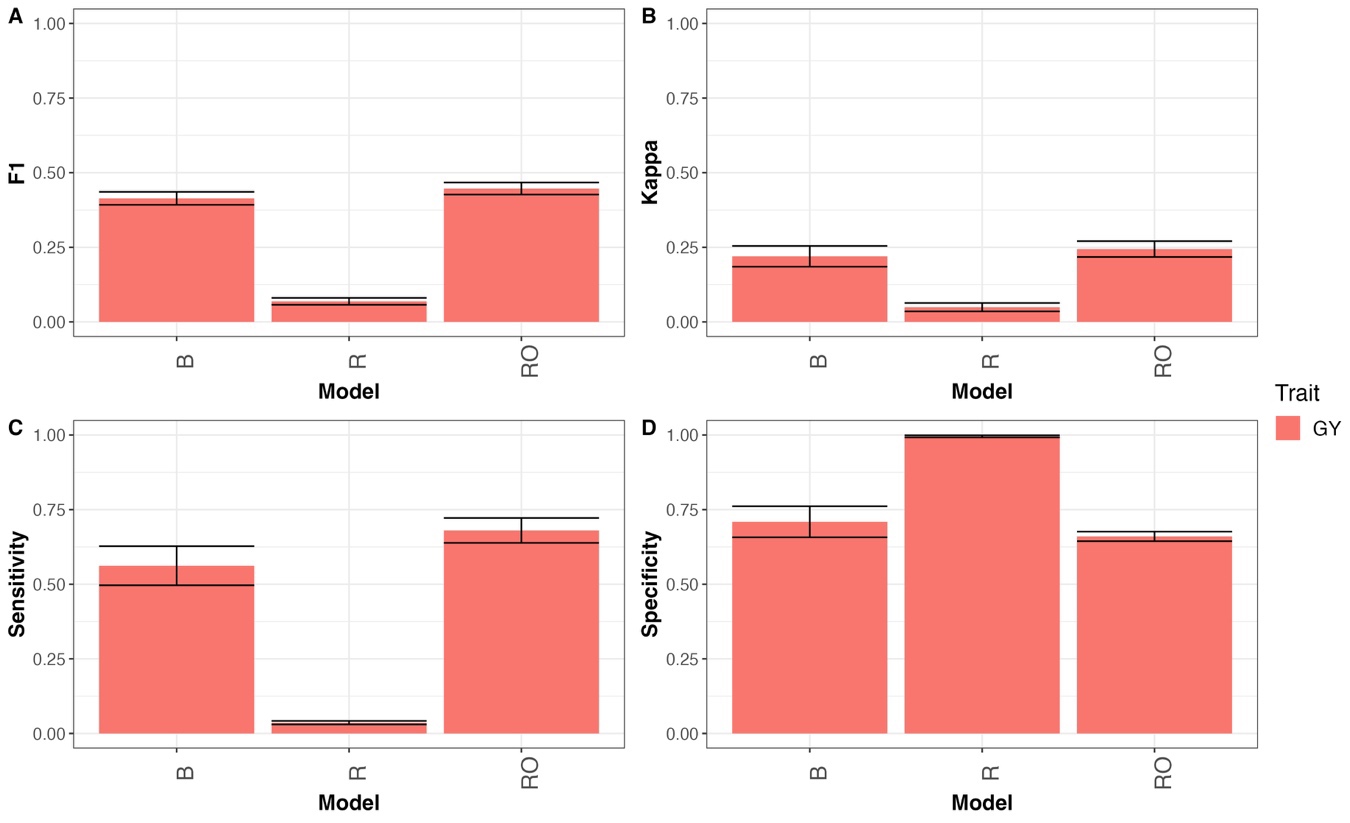


**Fig. S3 A)** Mean accuracy in terms of F1 score for classification model (B), regression model (R) and regression optimum model (RO) for trait GY for dataset Wheat_4. **B)** Mean accuracy in terms of Kappa coefficient for classification model(B), regression model (R) and regression optimum model (RO) for trait GY for dataset Wheat_4. **C)** Mean accuracy in terms of sensibility for classification model(B), regression model (R) and regression optimum model (RO) for trait GY for dataset Wheat_4. **D)** Mean accuracy in terms of specificity for classification model(B), regression model (R) and regression optimum model (RO) for trait GY for dataset Wheat_4.
